# Open-source LLMs for text annotation: a practical guide for model setting and fine-tuning

**DOI:** 10.1007/s42001-024-00345-9

**Published:** 2024-12-18

**Authors:** Meysam Alizadeh, Maël Kubli, Zeynab Samei, Shirin Dehghani, Mohammadmasiha Zahedivafa, Juan D. Bermeo, Maria Korobeynikova, Fabrizio Gilardi

**Affiliations:** 1 Department of Political Science, University of Zurich, 8050 Zurich, Switzerland; 2 Department of Computer Science, Institute for Fundamental Research, Tehran, Iran; 3 Department of Computer Engineering, Allameh Tabataba’i University, Tehran, Iran; 4 Department of Computer Science, Iran University of Science and Technology, Tehran, Iran

**Keywords:** ChatGPT, LLMs, Open source, FLAN, LLaMA, NLP, Text annotation

## Abstract

This paper studies the performance of open-source Large Language Models (LLMs) in text classification tasks typical for political science research. By examining tasks like stance, topic, and relevance classification, we aim to guide scholars in making informed decisions about their use of LLMs for text analysis and to establish a baseline performance benchmark that demonstrates the models’ effectiveness. Specifically, we conduct an assessment of both zero-shot and fine-tuned LLMs across a range of text annotation tasks using news articles and tweets datasets. Our analysis shows that fine-tuning improves the performance of open-source LLMs, allowing them to match or even surpass zero-shot GPT$-$3.5 and GPT-4, though still lagging behind fine-tuned GPT$-$3.5. We further establish that fine-tuning is preferable to few-shot training with a relatively modest quantity of annotated text. Our findings show that fine-tuned open-source LLMs can be effectively deployed in a broad spectrum of text annotation applications. We provide a Python notebook facilitating the application of LLMs in text annotation for other researchers.

**Supplementary Information:**

The online version contains supplementary material available at 10.1007/s42001-024-00345-9.

## Introduction

Generative Large Language Models (LLMs) such as GPT-3 and GPT-4 have demonstrated substantial potential for text-annotation tasks common to many Natural Language Processing (NLP) and political science applications [11]. Recent research reports impressive performance metrics for these models. For instance, studies demonstrate that GPT$-$3.5 exceeds the performance of crowd-workers in tasks encompassing relevance, stance, sentiment, topic identification, and frame detection [13], that it outperforms trained annotators in detecting the political party affiliations of Twitter users [37], and that it achieves accuracy scores over 0.6 for tasks such as stance, sentiment, hate speech detection, and bot identification [49]. Notably, GPT$-$3.5 also demonstrates the ability to correctly classify more than 70% of news as either true or false [15], which suggests that LLMs might potentially be used to assist content moderation processes.

While the performance of LLMs for text annotation is promising, several aspects remain unclear and require further research. Before proceeding to classify documents, the researcher must: (1) choose whether to employ an LLM without further training (zero-shot) or to manually annotate a subset of data and use a few-shot or fine-tuning strategy, (2) decide how many instances of data to annotate for few-shot or fine-tuning, (3) choose between GPT$-$3.5, GPT-4, and open-source LLMs such as LLaMA and FLAN. In the sections below, we offer empirical evidence demonstrating the significance of these decisions in practical terms and provide recommendations on how to best make their decisions. Throughout, our goal is to highlight the capabilities of open-source LLMs and fine-tuning approach, and provide a practical guide for researchers.

Zero-Shot Learning enables models to generalize to hitherto unseen tasks without the requirement for labeled examples, while Few-Shot Learning leverages a minimal set of annotated instances to adapt the model to new tasks. Despite their applicability, the conditions under which one paradigm outperforms the other remain an open question. On the other hand, fine-tuning constitutes the retraining of LLMs on a specialized, domain-specific dataset to augment task-specific performance. This process helps in endowing the LLM with domain-specific knowledge, while also potentially reducing some of the model biases. However, this may introduce biases present in the fine-tuning dataset, thereby requiring careful consideration of the data employed for this purpose. The extent to which Fine-Tuning and Few-Shot Learning methodologies substantively improve model performance remains indeterminate. Specifically, unresolved issues include the quantity of annotated data requisite for significant performance gains in the Fine-Tuning process, and whether Few-Shot Learning yields statistically meaningful improvements.

Moreover, the role of open-source LLMs deserves more attention. While models like GPT$-$3.5 have democratized the field by offering a more cost-effective alternative to traditionally more expensive annotation methods involving human annotations, open-source LLMs represent a further step towards greater accessibility. Beyond cost, the advantages of open-source LLMs include degrees of transparency and reproducibility that are typically not provided by commercial models. open-source LLMs can be scrutinized, tailored, and enhanced by a wider user base, fostering a diverse group of contributors and improving the overall quality and fairness of the models. Furthermore, open-source LLMs offer significant data protection benefits. They are designed not to share data with third parties, enhancing security and confidentiality. For these reasons, the academic community is increasingly advocating for the use of open-source LLMs [27, 36]. This transition would not only broaden researchers’ access to these tools but also promote a more open and reproducible research culture.

To address these questions, we extend previous research [13] to compare the performance of two widely-used open-source LLMs, LLaMA and FLAN, with that of GPT 3.5 as well as MTurkers, using eleven text annotation tasks distributed across four datasets. Each model is tested using different settings: varied model sizes for FLAN, and distinct temperature parameters in both zero-shot, few-shot, and fine-tuning approaches for GPT 3.5, LLaMA-1 (through HuggingChat), and LLaMA-2. We then compare their accuracy, using agreement with trained annotators as a metric, against that of MTurk as well as amongst themselves.

## Related work

The practice of fine-tuning transformer models for specialized tasks has become a cornerstone in the field of natural language processing (NLP). With the advent of large language models (LLMs) like GPT$-$3.5/4 and Bard (now Gemini), a burgeoning body of research is emerging to evaluate their performance and utility across various tasks.

LLMs have catalyzed a significant paradigmatic shift in the field of NLP, notably in the area of text annotation. Their capability to mimic human behavior, understand context, and adapt makes them effective for data annotation tasks [45]. This adaptability is primarily conditioned through intricate prompt engineering methods, ranging from zero-shot to few-shot promoting techniques, thereby focusing on generating accurate outputs [24].

Previous studies have extensively explored the capabilities of LLMs in diverse NLP applications such as text classification, text alignment, and semantic similarity tasks. [13] has shown that GPT$-$3.5 excels in multiple annotation tasks compared to human annotators. [38] further substantiates the efficacy of LLMs in the political arena by demonstrating that ChatGPT-4 outperforms both expert classifiers and crowd workers in classifying the political affiliation of Twitter posts, even when requiring reasoning based on contextual knowledge and author intentions. Promising results in named entity recognition, fact-checking and various text annotation tasks have also been reported [11, 12, 16]. However, the focus has largely been on practical NLP applications, leaving unexplored the potential in corpus pragmatics and corpus-assisted discourse studies [45].

However, it is imperative to acknowledge that the performance of LLMs is highly contingent upon both the quality of the dataset provided to be annotated and the proficiency of the model itself in this area [29]. Therefore, the ongoing refinement and fine-tuning of these models are imperative.

In terms of fine-tuning methodologies, various approaches have been developed. Notably, Parameter-Efficient Fine Tuning (PEFT) has garnered attention for optimizing LLMs in resource-constrained settings [20]. Another method that has received scholarly attention is the ’chain of thought’ technique, as validated by [43, 48]. The prevailing methodology, however, continues to be the attachment of task-specific heads to existing architectures, followed by domain-specific training and performance evaluation [18]. Moreover, several papers suggest that fine-tuning large language models can be effective in improving their performance and also reduce their size. [41] propose a structured pruning approach based on low-rank factorization and L0 norm regularization, which achieves significant inference speedups while maintaining or surpassing the performance of unstructured pruning methods. [5] explores the possibility of turning large language models into cognitive models by fine-tuning them on psychological experiment data, showing that they can accurately represent human behavior and outperform traditional cognitive models in decision-making tasks.

The ascendancy of proprietary LLMs has engendered a series of ethical and practical concerns, particularly pertaining to cost, transparency, and data protection. In contrast, open-source LLMs offer compelling advantages, including cost-effectiveness, methodological transparency, replicability, and stringent data protection standards [23, 27, 36].

Given the rapidly evolving landscape of LLM applications in text annotation and the critical role of prompt engineering in their performance, our work aims to evaluate the efficiency of fine-tuned open-source LLMs across a broader range of text annotation tasks. We also offer a side-by-side performance comparison with fine-tuned GPT$-$3.5.

## Materials and methods

### Data

The analysis relies on four distinct datasets. The first dataset consists of 2,978 randomly selected tweets from a more extensive collection of 2.6 million tweets related to content moderation, spanning from January 2020 to April 2021. The second dataset comprises 3,006 tweets posted by members of the US Congress between 2017 and 2022, sampled from a dataset of 20 million tweets. The third dataset consists of 2,480 newspaper articles on content moderation published from January 2020 to April 2021, drawn from a dataset of 980k articles obtained via LexisNexis. Sample sizes were determined based on the number of texts required to construct training sets for machine-learning classifiers. Finally, the fourth dataset replicates the data collection process of the first dataset. Specifically, it focused on January 2023, comprising a random sample of 1,313 Tweets from a dataset of 1.3 million tweets.

For the fine-tuning section of the three LLMs, we aim to allocate at least 15 % or more of the dataset to the evaluation set and the remaining 85 % to the training sets. We structure the training sets in increments of 50, 100, 250, 500, 1000, and 1500 samples, depending on the dataset’s size. If the evaluation contains fewer than 100 rows or less than two instances of the minority class (the least frequent class), we adjust its proportion upwards until these conditions are fulfilled. Beyond meeting these prerequisites, the evaluation set proportion is incrementally expanded by 5 % as long as it does not compromise the planned training set sizes. This approach aims to optimize the evaluation sample size across an array of training set dimensions, thereby enabling a comprehensive assessment of how varying training data volumes impact the performance of all three LLMs. This approach also facilitates a comparative analysis using identical datasets for zero-shot learning, few-shot learning, and fine-tuning (Table 1).

**Table 1:** Comprehensive Overview of Datasets Employed for Fine-Tuning Models Across Varied Tasks: Content Moderation Tweets from 2021 [A], Content Moderation Tweets from 2023 [B], Content Moderation News Articles from 2021 [C], and Tweets from the U.S. Congress spanning 2017 to 2021 [D]

Dataset	Task	Eval. Size	Fine-Tuning Size
A	Relevance	387	50,100,250,500,1000,1500
A	Problem/Solution	328	50,100,250,500
A	Policy Frames	843	50,100,250,500
A	Stance Detection	277	50,100,250,500,1000
A	Topics	307	50,100,250
B	Relevance	144	50,100,250,500,1000
B	Problem/Solution	100	50,100,250,500
C	Relevance	559	50,100,250,500,1000,1500
C	Problem Solution	196	50,100,250,500,1000
D	Relevance	836	50,100,250,500,1000,1500
D	Policy Frames	341	50,100,250,500

Specifications encompass Task Categories, Evaluation Dataset Dimensions, and Varied Sample Sizes Utilized in Fine-Tuning Assessment

### Data annotation tasks

We implemented several annotation tasks: (1) *relevance*: whether a tweet is about content moderation or, in a separate task, about politics; (2) *topic detection*: whether a tweet is about a set of six pre-defined topics (i.e. Section 230, Trump Ban, Complaint, Platform Policies, Twitter Support, and others); (3) *stance detection*: whether a tweet is in favor of, against, or neutral about repealing Section 230 (a piece of US legislation central to content moderation); (4) *general frame detection*: whether a tweet contains a set of two opposing frames (“problem’ and “solution”). The solution frame describes tweets framing content moderation as a solution to other issues (e.g., hate speech). The problem frame describes tweets framing content moderation as a problem on its own as well as to other issues (e.g., free speech); (5) *policy frame detection*: whether a tweet contains a set of fourteen policy frames proposed in [7]. The full text of instructions for the five annotation tasks is presented in Appendix S1. We used the exact same wordings for LLMs and MTurk.

### Trained annotators

We trained three political science students to conduct the annotation tasks. For each task, they were given the same set of instructions described above and detailed in Appendix S1. Importantly, to minimize inter-coder discrepancies and enhance the robustness of the annotation process, each of the students operated independently and systematically annotated the dataset task by task, adhering to a uniform codebook and shared procedural guidelines.

### Crowd-workers

To maintain a consistent comparative framework, we engaged workers from Amazon’s Mechanical Turk (MTurk) to execute the identical tasks administered to trained human annotators and Large Language Models (LLMs). These MTurk workers operated under the same instructional guidelines elaborated in Appendix S1. To ensure the quality and reliability of annotations, we imposed several restrictions on worker eligibility. Specifically, we limited task access to individuals designated as "MTurk Masters" by Amazon. Additionally, these workers were required to have a Human Intelligence Task (HIT) approval rate exceeding 90 % and a minimum of 50 approved HITs. We further restricted their geographic location to the United States. To mitigate the risk of undue influence from individual workers on the annotations for a specific task, we instituted a cap, ensuring that no single worker could contribute annotations to more than 20 % of the tweets allocated to a given task. Similar to our approach with trained human annotators, each tweet underwent annotation by two distinct MTurk workers to bolster the integrity and robustness of the collected data.

### LLM selection and settings

In our endeavor to evaluate the annotation performance and cost efficiency of various large language models (LLMs), we selected four distinct LLMs. The first model chosen was OPENAI’s GPT-4, GPT$-$3.5 (‘gpt$-$3.5-turbo’ version), a proprietary, closed-source LLM. Complementing this, we incorporated Meta’s LLaMA-1 (‘oasst-sft-6-LLaMA-30b’ version) and the more recent LLaMA-2 in two configurations: ‘LLaMA-2 13b’ and ‘LLaMA-2 70b as well as LLaMA-3 in its LLaMA-3 8b configuration. The selection was rounded off with FLAN-T5, a model we opted for due to its demonstrated promise in prior research [8, 50]. For FLAN-T5, available in sizes ranging from 80 M to 20B parameters, we experimented with the L, XL, and XXL variants to explore zero-shot capabilities (see Figure S1 in Appendix). Ultimately, we selected the FLAN-XL model for fine-tuning due to its advantageous balance of computational resource demands and text processing capabilities. This selection was driven by the model’s ability to provide a sophisticated understanding and processing of text, which is essential for optimal annotation performance.

In our Zero-Shot versus Few-Shot analysis, we employed only GPT$-$3.5 and LLaMA-1 (via HuggingChat) with the *temperature* set to 0.2. For the fine-tuning phase, we utilized the bare LLaMA-1 model, FLAN-XL, and other selected models, with the *temperature* set to 0.0. Our earlier findings informed this decision, where we observed a high level of agreement between runs with a *temperature* of 0.2, eliminating the need to run each model twice. Adopting a *temperature* of 0.0 for fine-tuning ensured the maximum level of output determinism, thereby enabling a more effective and efficient comparison between zero-shot and fine-tuned model performances.

### Prompt engineering

For zero-shot tests, we intentionally avoided adding any prompt engineering to ensure comparability between LLMs and MTurk crowd-workers. After testing several variations, we decided to feed tweets one by one to GPT$-$3.5 using the following prompt: “Here’s the tweet I picked, please label it as [Task Specific Instruction (e.g. ‘one of the topics in the instruction’)].” The corresponding prompts for each task are reported in Appendix S2. For few-shot tests, we employ Chain-of-Thought (CoT) prompting [43], where large language models (LLMs) are provided with both the question and a step-by-step reasoning answer as examples. Specifically, following previous research [21], we use GPT$-$3.5 to generate two CoT-prompted examples per class per annotation task. More specifically, we supplied GPT$-$3.5 with examples annotated by human experts, requesting an annotation and a substantiating explanation for the given annotation. Should the annotation provided by GPT$-$3.5 align with our human-generated labels-which serve as the ground truth-we subsequently incorporated both the example and GPT$-$3.5’s explanatory rationale into the prompt architecture for the few-shot learning experiment. Finally, we redeploy the zero-shot prompts in the fine-tuning phase to facilitate a comprehensive comparison between zero-shot and fine-tuned model performances.

### LLM fine-tuning

Pretraining Large Language Models (LLMs) on extensive corpora enables them to perform competently across a wide range of tasks with minimal examples, often achieving results that rival those of fine-tuned transformer models [6]. Specifically, in contexts where the LLM has not been sufficiently trained on task-relevant data, supervised fine-tuning can offer advantages. This involves supplementing the model with an additional dataset of labeled task-specific examples and selectively updating a subset of its weight parameters [28, 42]. However, while effective, fine-tuning such extensive models, particularly those with tens to hundreds of billions of parameters, can be computationally intensive, often requiring large-scale GPU clusters. However, recent advancements have made it feasible to fine-tune these models on single-GPU systems by employing techniques such as 4-bit or 8-bit quantization and adding lower-rank adapter layers to the original architecture [9].

In GPT$-$3.5, the fine-tuning process bridges the generalized learning acquired from pre-training and the specialized learning required for domain-specific Tasks. OpenAI’s GPT$-$3.5 architecture permits fine-tuning via its specialized Application Programming Interface (API), encompassing a multi-step workflow. Initially, the procedure necessitates the preparation of domain-specific datasets, generally constituting labeled instances. The transformation of this data involves segregating the input into three distinct components as mandated by the API. The first segment comprises the system prompt, articulating the overarching task instruction (e.g., Definition, Steps, Examples, etc.). Subsequently, the second segment encapsulates the user prompt, laden with domain-specific data and the instruction that requires labeling. The final segment incorporates the assistant prompt, directly indicating the target labels. To make sure the uploaded data adheres to the requested format, OpenAI provides a function to check for compatibility.^1^

Acting as a facilitative mechanism, the API enables users to delineate the training configuration, offering the liberty to customize hyperparameters, such as the number of epochs. Once the configuration is established and data uploaded, the API initiates the fine-tuning process. The model parameters are then updated iteratively to minimize the loss on the fine-tuning dataset. However, the inner workings of the fine-tuning process remain somewhat opaque, limiting interpretability and potential improvements. Post-fine-tuning, the model is evaluated on a held-out dataset to ascertain its performance on the target task. The fine-tuned model can then be deployed for the desired application.

We employed a combination of techniques to achieve efficient adaptation for fine-tuning the Open Source Models. Low-Rank Adaptation (LoRA) significantly reduces the number of trainable parameters by introducing low-rank matrices into each layer that capture task-specific adjustments [19]. Additionally, 4-bit quantization compresses the pre-trained model weights from 32-bit floating-point numbers to a more memory-efficient 4-bit representation, as described in [10]. This combination allows us to perform supervised fine-tuning on large models like FLAN-T5-xl [42], ‘oasst-sft-6-LLaMA-30b’ and ‘LLaMA-2 13b/70b’ [22] with better efficiency and potentially faster training times. We selected the xl-version of FLAN due to its enhanced capabilities relative to its smaller counterparts while keeping the computational demands reasonable. We used adapter layers on the Query and Value attention blocks in all three cases. As the training sets for each task are small and all are text classification tasks, we chose $r=16$ and $\alpha =32$ as hyperparameters for the adapter layers added. A lower rank was chosen to avoid overfitting to the training set, while the $\alpha$ was selected to produce a scaling of 2 and give more weight to the output of the adaptive layers and force the LLMs to follow the format used in the training set examples. As for the hyperparameters during training, we chose the default parameters of the Seq2SeqTrainer and SFTTrainer from huggingface [44].

To fine-tune ‘flan-t5-xl’, we used a single 80GB A100 GPU. The training examples had as input the zero-shot prompt with the coding or labeling guidelines followed by the text to label, and as output the letter that identified the label that should be assigned to the text (i.e: in the Relevance task ’A’ for relevant texts or ’B’ for irrelevant texts). For oasst-LLaMA, two 80GB A100 GPUs were needed to fine-tune the model. For the trainset examples, we followed the original prompt format used to fine-tune LLaMA-1 and produce the ‘oasst-sft-6-LLaMA-30b’ model^2^. In the segment designated for the prompter section, we reincorporated the zero-shot prompt and the corresponding text requiring labeling. Conversely, in the assistant section of the prompt, we included the target label to which the text should be mapped to. For LLaMA-2, we needed three 80GB A100 GPUs for the 70b model while only using one for the smaller 13b model. Furthermore, we maintained consistency with previous fine-tuning efforts by adhering to the original prompt format established for the "oasst-sft-6-LLaMA-30b" model. To optimize training efficiency, the input text for both models (LLaMA-1 & LLaMA-2) was left-truncated at a maximum of 4096 tokens. This combination of prompt design, targeted allocation of computational resources, and adherence to established prompting strategies facilitated effective fine-tuning of the LLaMA models. We trained all three LLMs for three epochs with a batch size of four. To further improve efficiency, we implemented a technique called gradient accumulation, where the model weights were only updated after accumulating gradients from every second batch.

### Evaluation metrics

We computed average accuracy (i.e. percentage of correct predictions), that is, the number of correctly classified instances over the total number of cases to be classified, using trained human annotations as our gold standard and considering only texts that both annotators agreed upon. Second, in applicable cases, we computed intercoder agreement, measured as the percentage of instances for which both annotators in a given group report the same class.

For the internal evaluation of the fine-tuning process, we employed additional metrics to garner more profound insights into the models’ learning and generalization across classes. Specifically, we calculated each class’s precision, recall, and F1 score to ensure that the fine-tuning process was comprehensive and aimed at enhancing performance across all classes. Precision is the ratio of correctly predicted positive observations to the total predicted positives, providing insight into the models’ ability to identify positive instances accurately. On the other hand, recall is the ratio of correctly predicted positive observations to all observations in actual class, shedding light on the models’ capability to identify all possible positive instances. The F1-score is the weighted average of precision and recall, thereby balancing the two metrics, especially in cases where one may have more significance than the other. Employing these metrics facilitated a robust evaluation, ensuring that the models were not biased towards the majority class and that the fine-tuning process effectively enhanced the models’ performance across all classes.

## Results

All results in this paper extend a previous study which compared GPT$-$3.5’s zero-shot annotation performance with that of MTurk [13]. We rely on extended datasets (n = 9,777), which include tweets and news articles that were collected and annotated manually on the discourse around content moderation [2], as well as a new sample of tweets posted in 2023 to address the concern that LLMs might be merely reproducing texts that could have been part of their training data. While the previous study used only GPT$-$3.5 for text classification, our analysis conducts the same classifications using GPT-4 as well as two open-source LLMs (LLaMA and FLAN), using the same codebook that was originally constructed for the research assistants and MTurkers (see S1).

### Choosing the training approach: zero-shot versus few-shot

Probably the first decision with respect to using LLMs for text annotation is whether to first manually annotate a subset of data and use it for few-shot learning or just directly proceed with a LLM in a zero-shot setting (see [6] for more background). Moreover, even if a researcher decides to have some manually annotated data, then the question is whether to use crowd-workers, or to recruit expert research assistants. The later question has already been answered in a previous study [13], in which the authors showed that GPT$-$3.5 outperforms crowd workers for several annotation tasks, including relevance, stance detection, topic modeling, and frame detection. Across the four datasets and a total of 12 annotation tasks, the zero-shot accuracy of GPT$-$3.5 exceeds that of crowd workers by about 25 percentage points on average.

For the purpose of answering the question of whether to go zero-shot or few-shot, before comparing these approaches, we would like to highlight the necessity of measuring the accuracy of LLMs with a priori annotated data. In fact, no matter how well GPT$-$3.5 or other LLMs have been reported to perform across various datasets and text annotation tasks, whenever a researcher is using a new dataset or need to implement a new annotation task, it is recommended to measure the accuracy of LLMs on a small subset of manually annotated data. The size of the test set varies in different papers (e.g. [13] and [50]). However, we recommend that a human expert manually annotate at least 100 data points.

**Fig. 1:**
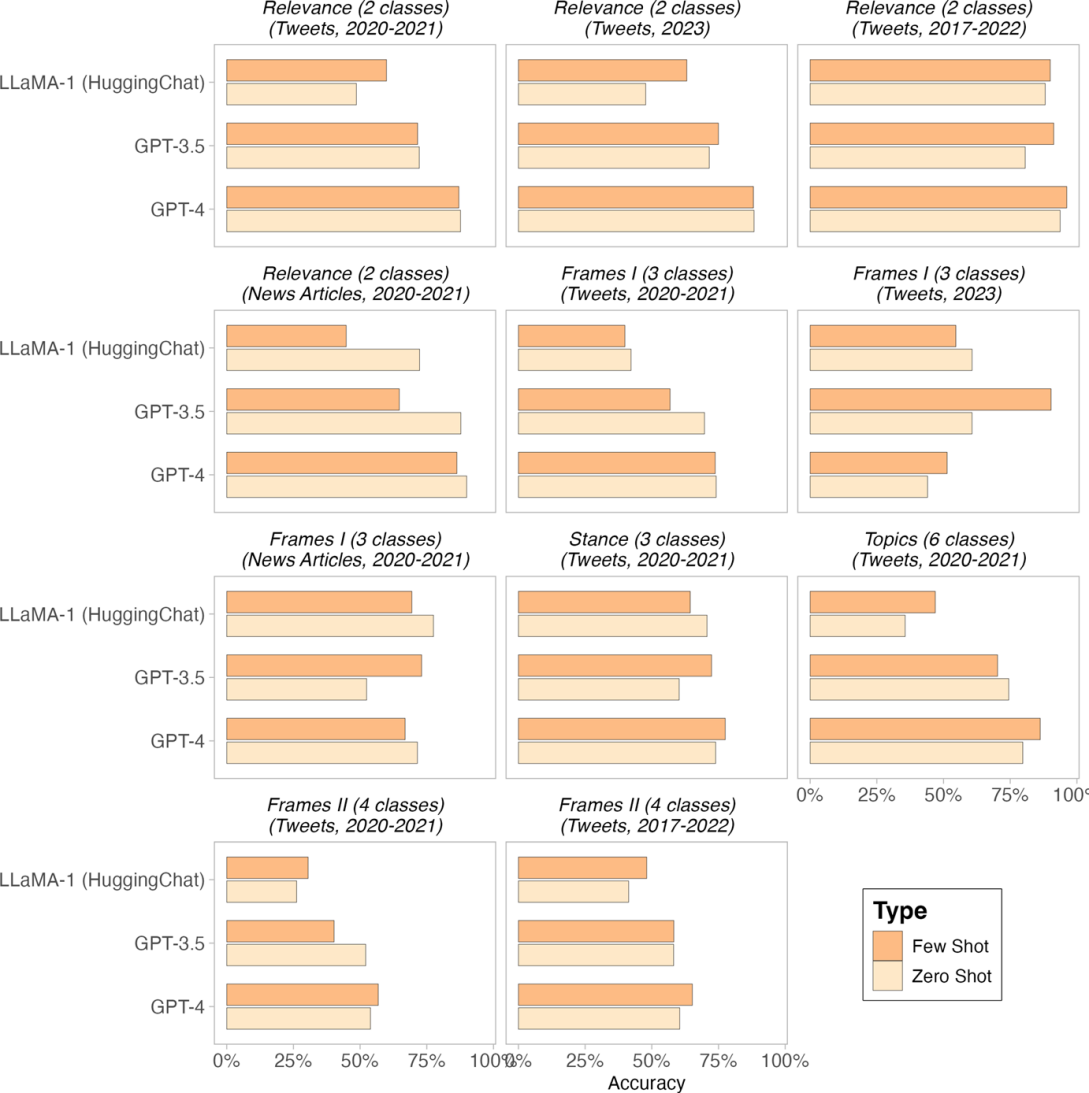
Comparing zero- and few-shot text annotation of GPT$-$3.5, GPT-4, and LLaMA-1 (HuggingChat). The x-axis shows the accuracy. The y-axis displays the two models grouped by the model configuration, including Zero-Shot and Few-Shot. Facets represent distinct tasks and/or datasets for evaluating model configurations

To understand whether it is safe for researchers to seamlessly use a LLM for text annotation in a zero-shot setting or not, here we extend the previous analysis of [13] to include few-shot learning. We conduct chain of thought (CoT) prompting [43]. This few-shot approach involves providing LLMs with question and step-by-step reasoning answer examples. In addition, we include results obtained from GPT-4 and HuggingChat (which uses LLaMA-1) as well. We chose HuggingChat due to its popularity and ease of use. The corresponding prompts are reported in S2. The results are illustrated in Fig. 1. Overall, in Fig. 1, we can see that the few-shot results are mixed, with some tasks slightly benefiting from few-shot learning, some performing lower, and some with no difference. For GPT-4 and GPT$-$3.5, we see performance gain for few-shot learning in 6 tasks and performance reduction in 5 tasks, though it varies across tasks. As for LLaMA-1 (HuggingChat), we see performance gain for few-shot learning in 4 tasks and performance reduction in 7 tasks.

With respect to what explains the mixed performance of few-shot learning across various text annotation tasks, we could not find any conclusive pattern. For example, let’s consider the number of classes as the measure for the complexity of the classification tasks. We see that the LLaMA-1 (HuggingChat) and GPT-4 benefited from few-shot learning in majority of more complex tasks, including topic modeling (6 classes), and framing detection (4 classes). However, GPT$-$3.5 saw a performance reduction in these three tasks. On the other hand, for the less complex tasks of relevance (first two plots from the top left in Fig. 1), we see that LLaMA-1 (HuggingChat) experienced significant performance gain from few-shot learning, but GPT-4 and GPT$-$3.5 results are mixed. As another example, exploring different types of datasets (tweets vs. news articles), we see that all GPT-4, GPT$-$3.5, and HuggingChat are showing less accuracy for few-shot learning in the relevance tasks, but in the framing detection task, the results are mixed, with GPT$-$3.5 benefiting from few-shot learning and GPT-4 and HuggingChat losing performance from it.

**Table:** 

Choosing a Training Approach: Zero- vs. Few-Shot	
**Advantages:**	
*Zero-Shot:* Off-the-shelf usage; no annotation cost; academic benchmarks on performance gain with few-shot learning are inconclusive.	
*Few-Shot:* Possibility of in-context learning with minimal examples; might lead to performance improvement.	
**Findings:**	
In our tests, few-shot results are mixed, some tasks benefited from it and some lost performance. No explicit pattern with respect to task complexity, model selection, and data type.	
**Advice:**	
Always manually annotate 100-250 data points to measure the accuracy of LLMs, specailly If working on a new dataset or task. We do not recommend spending much money and time on few-shot learning.	

### Temperature setting: higher versus lower

Another important decision that a researcher should make about using LLMs for text annotation is about the value of the temperature parameter. Both GPT$-$3.5 and LLaMA-1 (HuggingChat) have a temperature parameter which controls the degree of randomness, and thus the creativity, of the output. A higher temperature will result in more diverse and unexpected responses, while a lower temperature will result in more conservative and predictable responses. The default temperature value is 1.0 for GPT$-$3.5 and 0.9 for HuggingChat. Previous research showed that a lower temperature value may be preferable for text annotation tasks, as it seems to increase consistency without decreasing accuracy [13]. Here, we extend the previous research results by assessing the effect of a lower temperature in LLaMA-1 (HuggingChat). Similar to [13], we set the temperature at its default value and 0.2 and compare the outputs with respect to accuracy and intercoder agreement. We conducted two sets of annotations for each temperature value to compute LLM’s intercoder agreement.

Our results demonstrate that lower temperature settings significantly enhance intercoder agreement, underscoring the deterministic and repeatable nature of the annotations. For instance, GPT$-$3.5’s average intercoder agreement increased from 91.7% to 97.6% when the temperature was reduced from 1 to 0.2 in the zero-shot setting and from 92.3% to 95.4% in the few-shot setting. Similarly, for LLaMA-1 (HuggingChat), the agreement surged from 46.7% to 84.8% in the zero-shot and from 47.1% to 83.1% in the few-shot settings when the temperature was lowered from 0.9 to 0.2. These substantial improvements in intercoder agreement with lower temperatures provide a compelling case for their use in ensuring more deterministic and reliable LLM annotations. Moreover, our examination of accuracy values further supports the preference for lower temperature settings. Notably, the accuracy in the task of ’Stance’ classification on Tweets from 2020-2021 increased remarkably from 53.7% to 70.7% as the temperature lowered from 0.9 to 0.2. Additionally, in the ’Relevance’ classification for News Articles from 2020-2021, we observed a significant accuracy boost from 56.6% to 72.3% when the temperature setting was reduced. These examples underscore that a more deterministic approach, achieved by lowering the temperature, improves consistency and enhances the overall quality of results in various classification tasks, affirming the efficacy of employing lower temperature settings for improved LLM performance (Fig. 3).

**Fig. 2:**
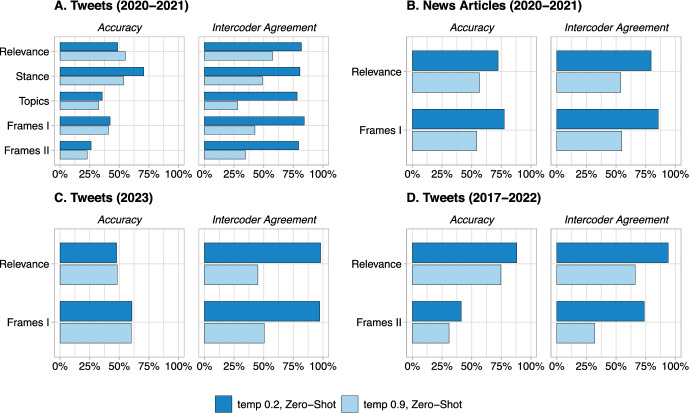
Analyzing the effect of LLaMA-1 (HuggingChat)’s temperature parameter on accuracy and intercoder agreement in text annotation tasks

**Fig. 3:**
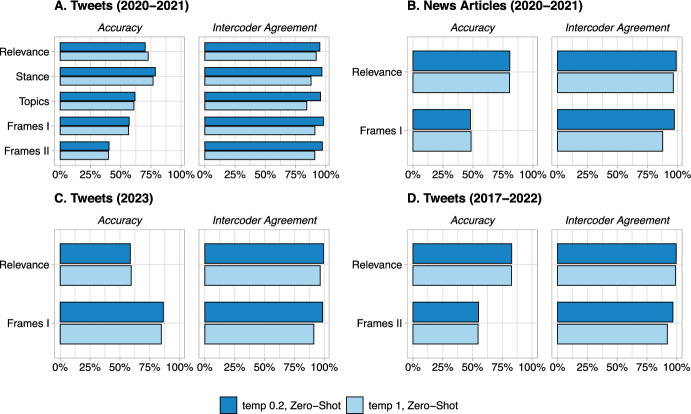
Analyzing the effect of GPT$-$3.5’s temperature parameter on accuracy and intercoder agreement in text annotation tasks

Across the four datasets, we report HuggingChat’s zero-shot performance for two different metrics: accuracy and intercoder agreement (Fig. 2). Accuracy is measured as the percentage of correct annotations (using our trained annotators as a benchmark), while the intercoder agreement is computed as the percentage of tweets that were assigned the same label by two different annotators (research assistant, crowd-workers, or GPT$-$3.5’s runs). Figure 2 shows that while the accuracy and intercoder agreement are, on average, lower than those reported for GPT$-$3.5 in [13], the pattern for the effect of temperature is the same. Across all four datasets and eleven annotation tasks, decreasing the temperature significantly improved the intercoder agreement scores without decreasing the accuracy. The only exception is for the relevance task in Tweet (2020-2021) dataset (first row in the top left plot in Fig. 2) which decreasing the temperature to 0.2 increased the intercoder agreement but led to reduction in accuracy. Interestingly, the GPT$-$3.5 results in 3 shows the same pattern for this particular task and dataset.

**Table:** 

Temperature Setting: High vs. Low	
**Advantages:**	
*Low Temperature:* Less randomness; less creative answers; more deterministic output.	
*High Temperature:* More randomness, more creative answers, more variations in outputs.	
**Findings:**	
Our analyses show that in almost all annotation tasks, lower temperature setting for GPT-3.5 and HuggingChat increases the intercoder agreement rate without decreasing the accuracy.	
**Advice:**	
We recommend setting the temperature parameter at zero (no randomness).	

### Model selection: proprietary versus open-source LLMs

Proprietary closed-source LLMs such as ChatGPT and Bard (Gemini) are more convenient and safe to use for general audiences due to their heavy fine-tuning to align with human preferences [36]. Although the training methodology is straightforward and simple, the extensive computational demands have restricted the creation of LLMs to a select few. That is why none of the open-source LLMs such as BLOOM, LLaMA-1 and Falcon could made a suitable substitutes for closed-source LLMs [39]. More recently, responding to this demand, LLaMA-2 and LLaMA-3 were introduced, which are a family of pretrained and fine-tuned LLMs at scales up to 70B parameters [39]. LLaMA-2 evaluations showed it outperforms LLaMA-1, Falcon, and MPT in standard academic benchmarks including commonsense reasoning, world knowledge, reading comprehension, and math, and performs on par with GPT$-$3.5 in math and popular aggregated benchmarks but fall short in Python code writing benchmarks [39].

Text annotation has always been costly. Although previous findings showed that GPT$-$3.5 outperforms Amazon Mechanical Turk crowd-workers (MTurker) and costs almost thirty times cheaper [13], it is not free of charge. Hence, it is tempting for researchers to explore the extent to which open-source LLMs are capable for text annotation tasks. In addition to cost-effectiveness, open-source LLMs are increasingly recognized for their transparency, reproducibility, and enhanced data protection features [27, 36]. However, the academic benchmarks reported above lack the text annotation tasks, especially that of political text. To assess how well open-source LLMs perform in text annotation tasks, we compare GPT (3.5 & 4) results with those of LLaMA (1 & 2), Llama-3 (8b) and FLAN (T5 XL). Considering the reported training data size, scaled-up parameters, and reading comprehension benchmarks, we expect the Llama-2 (70b) model to perform well on our text annotation tasks.

**Fig. 4:**
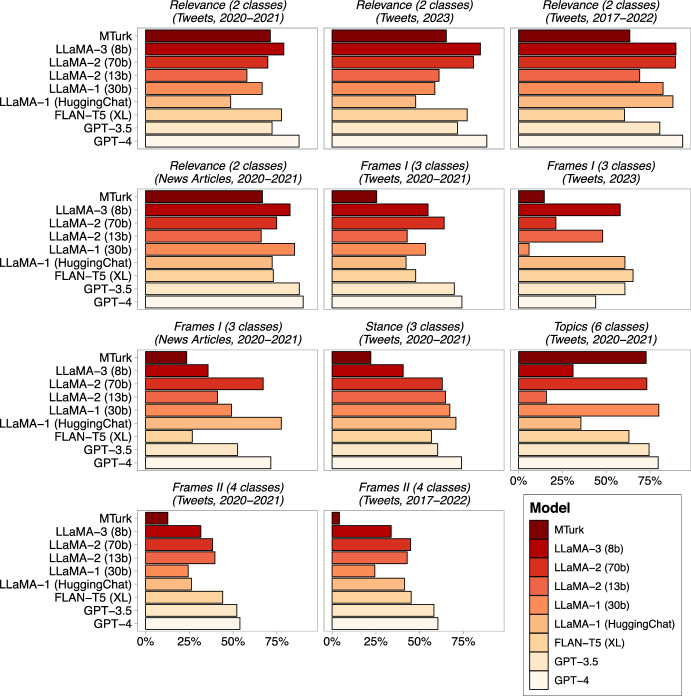
Accuracy of GPT$-$3.5, GPT-4, open-source LLMs, and MTurk. Accuracy means agreement with trained annotators. Bars indicate average accuracy, while whiskers range from minimum to maximum accuracy across models with different parameters and/or prompts (zero vs few shot)

Figure 4 compares the text annotation accuracy of GPT-4, GPT$-$3.5, LLaMA-1 (HuggingChat), LLaMA-1 (30b), LLaMA-2 (13b), LLaMA-2 (70b), Llama-3 (8b), FLAN-T5 (XL), and MTurkers. Seven observations stand out in this analysis: (1) no LLM outperforms others across all 11 annotation tasks; (2) only GPT-4 and GPT$-$3.5 outperform MTurkers in all 11 annotation tasks; (3) among the open-source LLMs, the best performing one in terms of the number of tasks that it outperforms MTurkers is LLaMA-2 (70b), with 9 out of 11 annotation tasks that it outperforms the crowd-workers; (4) among the open-source LLMs, the worst performing one in terms of the number of tasks that it outperforms MTurkers is LLaMA-2 (13b), with only 6 out of 11 annotation tasks that it outperforms the crowd-workers; (5) among the open-source LLMs, the best performing one in terms of outperforming GPT$-$3.5 is LLaMA-2 (70b), with 5 out of 11 annotation tasks it outperforms GPT$-$3.5; (6) Llama-3 (8b), which considers as a light LLM, performs on-par with GPT-4 on binary classification tasks, and outperform crowd-workers in 10 out of 11 tasks; (7) across the 9 annotation tasks that are related to datasets published before 2023, all open-source LLMs outperform crowd-workers where the number of classes is three and greater. In other words, when the data falls within an open-source LLM’s cutoff date and the annotation task is not a binary classification, all open-source LLMs perform better than MTurkers.

Overall, these findings underscore that while open-source LLMs are not consistently the superior choice, they generally outperform crowd-sourced annotations and are approaching the performance levels of GPT 3.5. Even an out-of-the-box tool such as HuggingChat, which looks and works very much like ChatGPT, outperforms Amazon Mechanical Turk crowd-workers in 8 out of all annotation tasks and performs close enough to GPT 3.5 in 6 out of 11 annotation tasks. While there is no universal answer to the question of what is the best open-source LLM for political text annotation, and the best performing LLM varies across dataset, task, and model size, our results show that a collection of open-source LLMs could almost perform on par with GPT$-$3.5. Therefore, we recommend to (1) use open-source LLMs for text annotation in social sciences research; and (2) compare the performance of 2-3 open-source LLMs, such as LLaMA-2 (70b), FLAN-T5 (XL), and LLaMA-1 (HuggingChat), and pick the best performing one.

**Table:** 

Model Selection: GPT-3.5/4 vs. Open-Source LLMs	
**Advantages:**	
*GPT-3.5/4:* Off-the-shelf usage; more convenient and safe to use; heavy fine-tuning to align with human preferences.	
*Open-Source LLMs:* No cost (GPT-3.5, and especially GPT-4 can become expensive for researchers without large research budgets); more transparency; ethical way to do research due to data privacy concerns; more reproducibility.	
**Findings:**	
Our results show that while open-source LLMs are not consistently the superior choice, they generally outperform crowd-sourced annotations and are approaching the performance levels of GPT 3.5 (ChatGPT).	
**Advice:**	
Use both LLaMA-2 (70b) and a light LLM from an easy-to-use open-source interface such as HuggingChat if resources allow for running heavy-size models, and pick the best-performing model. Use LLaMA-3 (8b) if high-performance computing is not available. Do not use LLaMA-2 (13b).	

### Annotation size: how much annotation is enough for fine-tuning?

In this section, we are interested in testing the effect of fine-tuning on the performance of both closed- and open-source LLMs. Advanced large language models (LLMs), such as GPT-4 and LLaMA-2, often demonstrate new capabilities and can learn from context with minimal examples, enabling them to perform complex tasks [35, 43]. However, fine-tuning these models is still necessary to unlock their full potential for creative and specialized tasks, aligning their performance with human preferences [34, 46]. Here, we would like to answer three important questions: (1) Would LLM exhibit performance gain in text annotation tasks when they get fine-tuned with human expert annotated data?; (2) If fine-tuning improves the performance of LLMs in text annotation accuracy, how big the training data should be?; and (3) Does the effect of fine-tuning on LLMs’ text annotation accuracy varies between close- and open-source LLMs?

Several factors may influence the efficacy of fine-tuning LLMs, including but not limited to 1) pretraining conditions; and 2) fine-tuning conditions. Pretraining factors include the size of the LLM and the volume of pretraining data, which are critical in determining the quality of the representation and knowledge encoded in the pretrained LLMs. On the other hand, fine-tuning conditions such as the nature of the downstream task, the size of the fine-tuning dataset, and the specific fine-tuning methodologies employed can significantly impact the extent of knowledge transfer to the targeted task [47]. Prior research has extensively investigated the scaling of LLM pretraining or training from scratch [17] as well as the development of advanced methods for fine-tuning [14]. However, the issue of whether and how the fine-tuning of LLMs scales with the fine-tuning conditions has been largely overlooked.

In this section, we are interested to explore the effect of LLM selection, LLM size, and the size of the fine-tuning data on the accuracy of LLMs on our running text annotation text. A recent study, based on three downstream tasks on translation and summarization, finds that the augmentation of the LLM model’s size exerts a more substantial influence on fine-tuning compared to increasing the size of fine-tuning data [47]. Furthermore, their results show that the effectiveness of fine-tuning varies across tasks and datasets, making the selection of the best fine-tuning approach for a particular downstream task less definitive. Considering these recent findings, we should expect to see a similar pattern, in which the performance gain from fine-tuning is being dependent on the text annotation task, model, model size, and the size of fine-tuning data.

**Fig. 5:**
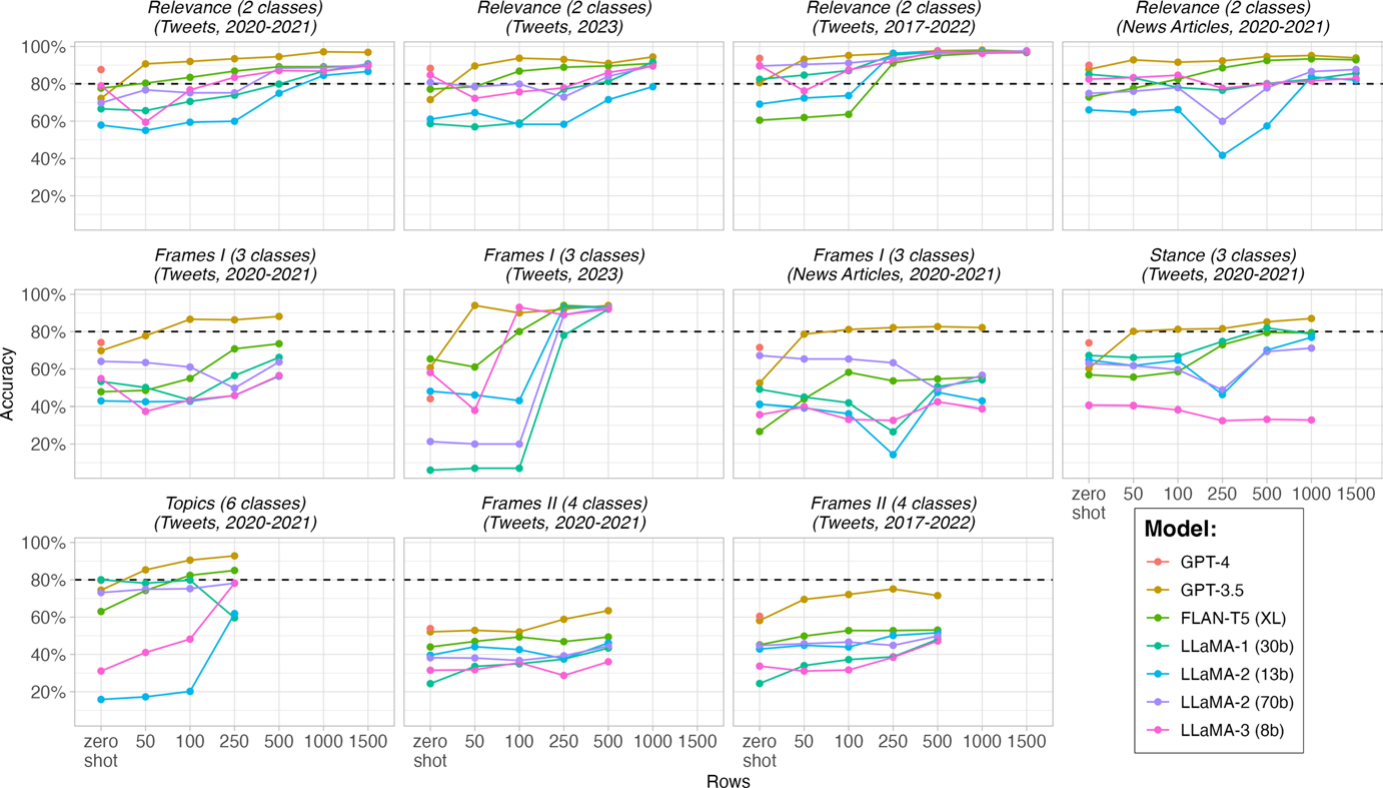
Performance (accuracy) of GPT$-$3.5, LLaMA-1, LLaMA-2, and FLAN-T5 (XL), as a function of the training data size for fine-tuning. The x-axis shows different sizes of training datasets, ranging from zero-shot (no fine-tuning) to 50, 100, 250, 500, and 1,000 rows used for fine-tuning the models. The y-axis displays the accuracy of the models in percentages. Facets represent distinct tasks and/or datasets for evaluating the models. Pink dots represent zero-shot GPT-4 accuracy for the sake of comparison

Figure 5 compares fine-tuning accuracy across five LLMs (LLaMA-1 (30b), LLaMA-2 (13b), LLaMA-2 (70b), FLAN-T5 (XL), and GPT 3.5), 11 text annotation tasks, between 4 to 7 different sizes of fine-tuning data (corresponding F1-scores are reported in Fig S2 in the Appendix). Several observations stand out in Fig. 5. First, with the exception of LLaMA-1 (30b), we see a general trend in other LLMs, in which the accuracy and F1-score of models improves with the size of fine-tuning data. Second, one of our salient findings in Fig. 5 and Figure S2 centers on the capabilities of FLAN-T5 XL. Our analysis reveals that, when fine-tuned, FLAN-T5 (XL) matches or even surpasses the zero-shot accuracy and F1-score of ChatGPT across all tasks and datasets, with a singular exception. It falls short in classifying the Problem-Solution frames in the 2023 tweets.

The third, and arguably the most compelling, finding in Fig. 5 and Fig. S2 centers on the differential rates and magnitudes of improvement observed across models during the fine-tuning process. ChatGPT demonstrates remarkable performance gains, even when fine-tuned with a minimal dataset of just 50 instances. Specifically, it registers an average accuracy increase of 15.7 %, which further escalates to 19.1 % when the training set comprises 100 cases. In a stark contrast, open-source models such as FLAN-T5 (XL) and LLaMA-1 exhibit a more incremental progression in performance. Intriguingly, the LLaMA-1 model initially sees a dip in accuracy on certain tasks but tends to recover and improve as the dataset expands to around 250 instances. By this point, both FLAN-T5 (XL) and LLaMA-1 come close to matching the zero-shot accuracy levels achieved by ChatGPT. It is worth noting that the average accuracy gains for FLAN-T5 (XL) stand at 7.7 % and 12.4 % when fine-tuned with 50 and 100 instances, respectively. This underscores the point that open-source models, too, stand to benefit significantly from fine-tuning.

These findings illuminate the complex dynamics at play in the fine-tuning of LLMs for text annotation tasks. They highlight the variable performance across different models and tasks, the rapid yet plateauing gains for commercial models like ChatGPT, and the more gradual but sustained improvements for open-source models. Next, fine-tuning is particularly helpful when using GPT$-$3.5 Turbo, even with as little as 50 rows of training data. The results are mixed for open-source models like FLAN-T5 (XL) and LLaMA-1. FLAN-T5 (XL) generally benefits from fine-tuning, while LLaMA-1’s performance is inconsistent. However, as shown in Fig. 5, increasing the amount of training data does improve LLaMA-1’s performance over its zero-shot capabilities, just like for all other models.

In our study, we observed that the cost of fine-tuning commercial models like ChatGPT is quite reasonable. The total expenditure for fine-tuning all our ChatGPT models was only $311, with an additional $34 for the evaluation of these models. Specifically, fine-tuning GPT$$-$$3.5 costs merely $1.2 for 100 rows across three epochs, with subsequent usage costs being only 16 Cents per 100 rows. These figures illustrate that even when relying on commercial models, fine-tuning is an economically viable option, especially when compared to the costs of zero-shot usage. While open-source LLMs can offer significant cost savings when suitable infrastructure is available, fine-tuning commercial models like ChatGPT remains an affordable and efficient alternative for those without access to such resources.

In summary, our empirical findings robustly advocate for fine-tuning as the primary strategy for enhancing classification accuracy across varying tasks and models. Although zero-shot and few-shot learning paradigms may offer utility under specific circumstances, fine-tuning emerges as the most consistently effective approach. However, it is important to note that the efficacy of fine-tuning is not universally high across all task complexities. Specifically, the availability of substantial, high-quality training datasets becomes imperative for achieving optimal performance levels for intricate classification tasks. Despite fine-tuning, it remains possible that the model’s performance may not meet the thresholds required for specific specialized applications. This is particularly relevant as more labeled datasets become available, making fine-tuning a practical choice for those aiming to optimize classification tasks.

**Table:** 

Annotation Size: How much annotation is enough for fine-tuning?	
**Advantages:**	
*50-100 Manual Annotation:* Less cost; less time consuming.	
*250-500 Manual Annotation:* Potential for higher performance gain.	
**Findings:**	
In general, LLMs’ accuracy increases with the size of fine-tuning data. FLAN-T5 (XL) matches or even surpasses GPT-3.5’s zero-shot performance across all tasks and datasets (except one task). Open-source LLMs and GPT-3.5 differ in the optimal number of required fine-tuning data.	
**Advice:**	
Our empirical findings robustly advocate for fine-tuning as the primary strategy for enhancing classification accuracy. If using GPT-3.5, 50 annotated data points are enough. If using an open-source LLM, go with 250.	

### A note on explainability of LLMs performance

Throughout the paper, we presented some mixed results about which LLM outperforms others in each annotation task. For example, for comparison between zero- and few-shot annotation, we witnessed very mixed results with no universal pattern explaining what drives the performance differences. We anticipate that various factors will contribute to explaining the behaviors and capabilities of large language models (LLMs) in text annotation tasks. These factors include but are not limited to, the topic at hand and the model’s knowledge of it, the cut-off date for the model’s training data, the quality and diversity of the data used in training, the complexity of the tasks presented, the inherent stochasticity of LLMs, and the extent of fine-tuning applied by the developers after pre-training. several notes are of importance here:

1. Capabilities vs. Behaviors: LLMs’ capabilities stem from the pre-training stage, which is a resource-intensive and often static process. In contrast, their behavior, particularly in tasks such as question answering, is shaped by fine-tuning, which is more cost-effective and can be updated more frequently [26].
2. Transparency and Interpretability: In many cases, there is limited transparency regarding the specific data on which LLLMs have been trained, both in terms of the textual corpus utilized during pre-training and the supplementary instructions provided in fine-tuning stage. This opacity presents significant challenges in explaining why a particular input yields a specific output, particularly in relation to the model’s internal weight structure and the training data employed. Consequently, we advise researchers to exercise caution when employing these models in domains where interpretability and explainability are critical [32].
3. Unintended consequences of Fine-Tuning: Fien Tuning can sometimes lead to unintended performance degradation or improvements, which developers constantly work to address [26].
4. Nondeterministic Behaviors: The combination of fine-tuning and the inherent nondeterminism in LLMs can result in unpredictable outcomes. For example, it has been shown that GPT-4, when asked to find prime numbers in a given set, may skip part of the reasoning process, failing to thoroughly evaluate each number. Such behavior could similarly occur in our experiments, potentially contributing to the mixed results we observed [26].
5. It is shown that the topic of the conversation has sometimes induce a significant effect on the performance of the LLMs [1]. It is possible that if we expand the diversity of the topics, we could find a pattern on when and why we see some mixed results and why LLMs produce what the produce in text annotation.

A potential approach for assessing the impact of the aforementioned factors influencing the LLMs is the use of the ‘integrated gradients’ method [33]. This technique focuses on identifying the most influential input tokens rather than exhaustively considering all possible factors. However, as the primary objective of this paper is to offer practical guidelines for social science researchers on initiating the use of LLMs for text annotation tasks, as well as to establish a baseline performance benchmark that demonstrates the models’ effectiveness in this domain, we reserve this experiment for future research.

## Conclusion

Automated classification of short and long text is central to a growing number of research questions in the social sciences. Previous research advocated for using supervised machine learning methods over dictionary-based approaches and provided best practices for human annotation of text [4]. However, the emergence of large language models (LLM) and their ability to outperform crowd-workers in text annotation and yielding acceptable accuracy compared to human expert evaluation [13] provide researchers with new opportunities to skip the crowd-sourcing or even training their own supervised machine learning models for text classification. Nevertheless, in the rush to take advantage of these opportunities, one can easily neglect to consider crucial questions and underestimate the implications of certain choices.

In this paper, we have tried to walk the researchers through critical decisions they need to make for using LLMs in text annotation tasks (e.g. relevance, topic detection, and framing detection) and provided them with some practical advice backed by our empirical results. Our most surprising finding is the substantial effect of fine-tuning on increasing LLMs text annotation performance. We demonstrate that open-source LLMs such as LLaMA-1, LLaMA-2, and FLAN represent a competitive alternative for text annotation tasks, exhibiting performance metrics that generally exceed those of Amazon Mechanical Turk crowd-workers and rival those of GPT 3.5 (ChatGPT). An important appeal of open-source LLMs is that they offer considerable cost advantages. While ChatGPT provides substantial cost-efficiency, being about thirty times more affordable per annotation compared to MTurk [13], open-source LLMs surpass this by being freely available. This constitutes a significant improvement in the accessibility of such models, extending their reach to a broader range of researchers irrespective of financial constraints.

Open-source LLMs present benefits that go beyond cost-efficiency. One key advantage is that they help reduce reliance on proprietary models operated by for-profit companies, which may conflict with research ethics and the reproducibility standards [27, 36]. Furthermore, open-source LLMs provide distinct benefits for data protection, as they are designed in such a way that data do not need to be shared with any third-party entities [40]. This feature ensures that sensitive information remains secure and confidential because it is not sent to or stored by an external party. The elimination of data sharing in open-source LLMs provides an extra layer of protection against potential data breaches or unauthorized access. This feature becomes especially beneficial in scenarios where sensitive data is involved, such as in the legal or medical fields, where confidentiality is of utmost importance [3, 30, 31], but also in social science research involving data protected under the European Union’s General Data Protection Regulation (GDPR), or covered by non-disclosure agreements (NDAs) [25].

We conclude with four general pieces of advice for text analysts: (1) manually annotate 250-500 data points and use half for fine-tuning and half for accuracy testing; (2) use fine-tuned open-source LLMs for text annotation due to their cost-effectiveness, transparency, and reproducibility; (3) always validate the output of LLMs by human expert evaluation; (4) run LLMs at least twice per task and report average accuracy and intercoder agreement; and (5) set the temperature of GPT or LlaMA models at zero or a low value to get higher intercoder agreement without loss in accuracy.

While the findings presented in this study regarding the performance of open-source large language models (LLMs) in text annotation tasks are encouraging, it is crucial to underscore that these results should not be broadly generalized across all forms of text annotation tasks or datasets. The specificity of the tasks and datasets used in our experiments may not fully represent the diversity of potential use cases in other research contexts. Therefore, we strongly recommend that researchers exercise caution when drawing conclusions from our results and conduct their own empirical evaluations. This is particularly important when engaging with distinct datasets or addressing novel annotation tasks, as variations in data characteristics or task complexities may significantly influence the performance of LLMs. The decisions and recommendations discussed in this paper should be viewed as a framework for further experimentation, rather than definitive guidance applicable to all annotation scenarios.

## Supplementary Information

Below is the link to the electronic supplementary material.

Supplementary file 1 (pdf 315 KB)

## Data Availability

As part of our commitment to transparency and reproducibility, we have made all the necessary files available to replicate the analyses presented in this manuscript. The replication package includes datasets, jupyter Python notebook for using and fine-tuning open-source LLMs, and additional supplementary materials used in our study. The replication files can be accessed at the following URL: https://osf.io/ctgqx/.

## Footnotes

1 Additional information for Data preparation and analysis for chat model fine-tuning can be found here: https://cookbook.openai.com/examples/chat_finetuning_data_prep.

2 https://github.com/LAION-AI/Open-Assistant/blob/main/model/MESSAGE_AND_TOKEN_FORMAT.md.
