# Supplementary material for: Open-source LLMs for text annotation: a practical guide for model setting and fine-tuning
Source: J Comput Soc Sci. 2024 Dec 18;8(1):17. doi: 10.1007/s42001-024-00345-9 (PMC11655591; doi:10.1007/s42001-024-00345-9)
Supplement: Supplementary file 1 — (pdf 315 KB) [file 42001_2024_345_MOESM1_ESM.pdf]

S1. Zero-Shot Annotation Codebook

S1.1 Dataset 1: Content Moderation Tweets (2020-2021)

S1.1.1 Task 1: Relevance

"Content moderation" refers to the practice of screening and monitoring content posted by users on social media sites to determine if the content should be published or not, based on specific rules and guidelines.

I will ask you to classify a text as relevant or irrelevant to the content moderation:

A: Text is RELEVANT if it includes: social media platforms' content moderation rules and practices, censorship, governments' regulation of online content moderation, and/or mild forms of content moderation like flagging, shadowbanning, or account suspension.

B: Text is IRRELEVANT if they do not refer to content moderation, as defined above. This would include, for example, a tweet by Trump that Twitter has labeled his tweet as "disputed", or a tweet claiming that something is false.

Now, is the following text relevant or irrelevant to content moderation?

[Paste a tweet here and remove the brackets]

S1.1.2 Task 2: Problem/Solution Frames

"Content moderation" refers to the practice of screening and monitoring content posted by users on social media sites to determine if the content should be published or not, based on specific rules and guidelines.

I will ask you to classify a text as describing content moderation as a problem, as a solution, or neither:

A: Text describes content moderation as a PROBLEM if they emphasize negative effects of it, such as restrictions to free speech, censorship, or the biases that can emerge from decisions regarding what users are allowed to post.

B: Text describes content moderation as a SOLUTION if they emphasize positive effects of it, such as protecting users from harmful content such as hate speech, misinformation, illegal adult content, or spam.

C: Text describes content moderation as NEUTRAL if they do not emphasize negative or positive effects of content moderation. For example if they simply report on the content moderation activity of social media platforms without linking them to potential advantages or disadvantages for users or stakeholders.

Now, is the following text describing content moderation as a problem, as a solution, or neither?

[Paste a tweet here and remove the brackets]

S1.1.3 Task 3: Policy Frames

"Content moderation" refers to the practice of screening and monitoring content posted by users on social media sites to determine if the content should be published or not, based on specific rules and guidelines.

I will ask you to classify a text as one of the frames defined below:

- ECONOMY: The costs, benefits, or monetary/financial implications of the issue (to an individual, family, community, or to the economy as a whole).
- Capacity and resources: The lack of or availability of physical, geographical, spatial, human, and financial resources, or the capacity of existing systems and resources to implement or carry out policy goals.

- MORALITY: Any perspective—or policy objective or action (including proposed action)that is compelled by religious doctrine or interpretation, duty, honor, righteousness or any other sense of ethics or social responsibility.
- FAIRNESS AND EQUALITY: Equality or inequality with which laws, punishment, rewards, and resources are applied or distributed among individuals or groups. Also the balance between the rights or interests of one individual or group compared to another individual or group.
- POLICY PRESCRIPTION AND EVALUATION: Particular policies proposed for addressing an identified problem, and figuring out if certain policies will work, or if existing policies are effective.
- LAW AND ORDER, CRIME AND JUSTICE: Specific policies in practice and their enforcement, incentives, and implications. Includes stories about enforcement and interpretation of laws by individuals and law enforcement, breaking laws, loopholes, fines, sentencing and punishment. Increases or reductions in crime.
- SECURITY AND DEFENSE: Security, threats to security, and protection of one's person, family, in-group, nation, etc. Generally an action or a call to action that can be taken to protect the welfare of a person, group, nation sometimes from a not yet manifested threat.
- HEALTH AND SAFETY: Health care access and effectiveness, illness, disease, sanitation, obesity, mental health effects, prevention of or perpetuation of gun violence, infrastructure and building safety.
- QUALITY OF LIFE: The effects of a policy on individuals' wealth, mobility, access to resources, happiness, social structures, ease of day-to-day routines, quality of community life, etc.
- POLITICAL: Any political considerations surrounding an issue. Issue actions or efforts or stances that are political, such as partisan filibusters, lobbyist involvement, bipartisan efforts, deal-making and vote trading, appealing to one's base, mentions of political maneuvering. Explicit statements that a policy issue is good or bad for a particular political party.
- EXTERNAL REGULATION AND REPUTATION: The United States' external relations with another nation; the external relations of one state with another; or relations between groups. This includes trade agreements and outcomes, comparisons of policy outcomes or desired policy outcomes.
- OTHER: Any topic that does not fit into the above categories.

Now, which of the above frames best fit the following text? Answer with only the option above that is most accurate and nothing else.

[Paste a tweet here and remove the brackets]

S1.1.4 Task 4: Stance Detection

"Content moderation" refers to the practice of screening and monitoring content posted by users on social media sites to determine if the content should be published or not, based on specific rules and guidelines. In the context of content moderation, Section 230 is a law in the United States that protects websites and other online platforms from being held legally responsible for the content posted by their users. This means that if someone posts something illegal or harmful on a website, the website itself cannot be sued for allowing it to be posted. However, websites can still choose to moderate content and remove anything that violates their own policies.

I will ask you to classify a text as in favor of, against, or neutral about Section 230:

A. "In favor of" expresses approval for Section 230 and/or advocates keeping Section 230
B. "Against" expresses disapproval towards Section 230 and/or advocates repealing Section 230
C. "Neutral" discusses Section 230 without expressing approval or disapproval towards it

Now, is the following text in favor of, against, or neutral about Section 230?
[Paste a tweet here and remove the brackets]

S1.1.5 Task 5: Topic Detection

"Content moderation" refers to the practice of screening and monitoring content posted by users on social media sites to determine if the content should be published or not, based on specific rules and guidelines.

I will ask you to classify a text as of the topics described below:

1. Section 230, which is a law in the United States that protects websites and other online platforms from being held legally responsible for the content posted by their users (SECTION 230).
2. The decision by many social media platforms, such as Twitter and Facebook, to suspend Donald Trump's account (TRUMP BAN).
3. Requests directed to Twitter's support account or help center (TWITTER SUPPORT).
4. Social media platforms' policies and practices, such as community guidelines or terms of service (PLATFORM POLICIES).
5. Complaints about platform's policy and practices in deplatforming and content moderation or suggestions to suspend particular accounts, or complaints about accounts being suspended or reported (COMPLAINTS).
6. If a text is not about the SECTION 230, COMPLAINTS, TRUMP BAN, TWITTER SUPPORT, and PLATFORM POLICIES, then it should be classified in OTHER class (OTHER).

Now, is the following text about SECTION 230, TRUMP BAN, COMPLAINTS, TWITTER SUPPORT, PLATFORM POLICIES, or OTHER?
[Paste a tweet here and remove the brackets]

S1.2 Dataset 2: Content Moderation Tweets (2023)

S1.2.1 Task 1: Relevance

"Content moderation" refers to the practice of screening and monitoring content posted by users on social media sites to determine if the content should be published or not, based on specific rules and guidelines.

I will ask you to classify a text as relevant or irrelevant to the content moderation:

A: Text is RELEVANT if it includes: social media platforms' content moderation rules and practices, censorship, governments' regulation of online content moderation, and/or mild forms of content moderation like flagging, shadowbanning, or account suspension.

B: Text is IRRELEVANT if they do not refer to content moderation, as defined above. This would include, for example, a tweet by Trump that Twitter has labeled his tweet as "disputed", or a tweet claiming that something is false.

Now, is the following text relevant or irrelevant to content moderation?
[Paste a tweet here and remove the brackets]

S1.2.2 Task 2: Problem/Solution Frames

"Content moderation" refers to the practice of screening and monitoring content posted by users on social media sites to determine if the content should be published or not, based on specific rules and guidelines.

I will ask you to classify a text as describing content moderation as a problem, as a solution, or neither:

A: Text describes content moderation as a PROBLEM if they emphasize negative effects of it, such as restrictions to free speech, censorship, or the biases that can emerge from decisions regarding what users are allowed to post.

B: Text describes content moderation as a SOLUTION if they emphasize positive effects of it, such as protecting users from harmful content such as hate speech, misinformation, illegal adult content, or spam.

C: Text describes content moderation as NEUTRAL if they do not emphasize negative or positive effects of content moderation. For example if they simply report on the content moderation activity of social media platforms without linking them to potential advantages or disadvantages for users or stakeholders.

Now, is the following text describing content moderation as a problem, as a solution, or neither?

[Paste a tweet here and remove the brackets]

S1.3 Dataset 3: US Congress Members Tweets (2017-2022)

S1.3.1 Task 1: Relevance

"Political content" refers to a text that pertains to politics or government policies at the local, national, or international level. This can include political figures, events, or issues, as well as text that uses political language or hashtags.

I will ask you to classify a text as relevant or irrelevant to the political content:

Text is relevant if it uses political keywords or hashtags, mentions political figures or events, discusses policy issues such as immigration, abortion, foreign policy, health care, tax, or police shootings, or includes a link to news outlets or other political sources such as think tanks, political pundits or journalists, the White House, or the US Congress. Text is irrelevant if it does not fit the criteria above

Now, is the following text relevant or irrelevant to political content?

[Paste a tweet here and remove the brackets]

S1.3.2 Task 2: Policy Frames

"Political content" refers to a text that pertains to politics or government policies at the local, national, or international level. This can include political figures, events, or issues, as well as text that uses political language or hashtags.

I will ask you to classify a text as one of the frames defined below:

- ECONOMY: The costs, benefits, or monetary/financial implications of the issue (to an individual, family, community, or to the economy as a whole).
- Capacity and resources: The lack of or availability of physical, geographical, spatial, human, and financial resources, or the capacity of existing systems and resources to implement or carry out policy goals.
- MORALITY: Any perspective—or policy objective or action (including proposed action)that is compelled by religious doctrine or interpretation, duty, honor, righteousness or any other sense of ethics or social responsibility.
- FAIRNESS AND EQUALITY: Equality or inequality with which laws, punishment, rewards, and resources are applied or distributed among individuals or groups. Also the balance between the rights or interests of one individual or group compared to another individual or group.

- POLICY PRESCRIPTION AND EVALUATION: Particular policies proposed for addressing an identified problem, and figuring out if certain policies will work, or if existing policies are effective.
- LAW AND ORDER, CRIME AND JUSTICE: Specific policies in practice and their enforcement, incentives, and implications. Includes stories about enforcement and interpretation of laws by individuals and law enforcement, breaking laws, loopholes, fines, sentencing and punishment. Increases or reductions in crime.
- SECURITY AND DEFENSE: Security, threats to security, and protection of one's person, family, in-group, nation, etc. Generally an action or a call to action that can be taken to protect the welfare of a person, group, nation sometimes from a not yet manifested threat.
- HEALTH AND SAFETY: Health care access and effectiveness, illness, disease, sanitation, obesity, mental health effects, prevention of or perpetuation of gun violence, infrastructure and building safety.
- QUALITY OF LIFE: The effects of a policy on individuals' wealth, mobility, access to resources, happiness, social structures, ease of day-to-day routines, quality of community life, etc.
- POLITICAL: Any political considerations surrounding an issue. Issue actions or efforts or stances that are political, such as partisan filibusters, lobbyist involvement, bipartisan efforts, deal-making and vote trading, appealing to one's base, mentions of political maneuvering. Explicit statements that a policy issue is good or bad for a particular political party.
- EXTERNAL REGULATION AND REPUTATION: The United States' external relations with another nation; the external relations of one state with another; or relations between groups. This includes trade agreements and outcomes, comparisons of policy outcomes or desired policy outcomes.
- OTHER: Any topic that does not fit into the above categories.

Now, which of the above frames best fit the following text? Answer with only the option above that is most accurate and nothing else.

[Paste a tweet here and remove the brackets]

S1.4 Dataset 4: Content Moderation News Articles (2020-2021)

S1.4.1 Task 1: Relevance

"Content moderation" refers to the practice of screening and monitoring content posted by users on social media sites to determine if the content should be published or not, based on specific rules and guidelines.

I will ask you to classify a text as relevant or irrelevant to the content moderation:

A: Text is RELEVANT if it includes: social media platforms' content moderation rules and practices, censorship, governments' regulation of online content moderation, and/or mild forms of content moderation like flagging, shadowbanning, or account suspension.

B: Text is IRRELEVANT if they do not refer to content moderation, as defined above. This would include, for example, a tweet by Trump that Twitter has labeled his tweet as "disputed", or a tweet claiming that something is false.

Now, is the following text relevant or irrelevant to content moderation?

[Paste a news article text here and remove the brackets]

S1.4.2 Task 2: Problem/Solution Frames

"Content moderation" refers to the practice of screening and monitoring content posted by users on social media sites to determine if the content should be published or not, based on specific rules and guidelines.

I will ask you to classify a text as describing content moderation as a problem, as a solution, or neither:

A: Text describes content moderation as a PROBLEM if they emphasize negative effects of it, such as restrictions to free speech, censorship, or the biases that can emerge from decisions regarding what users are allowed to post.

B: Text describes content moderation as a SOLUTION if they emphasize positive effects of it, such as protecting users from harmful content such as hate speech, misinformation, illegal adult content, or spam.

C: Text describes content moderation as NEUTRAL if they do not emphasize negative or positive effects of content moderation. For example if they simply report on the content moderation activity of social media platforms without linking them to potential advantages or disadvantages for users or stakeholders.

Now, is the following text describing content moderation as a problem, as a solution, or neither?

[Paste a news article text here and remove the brackets]

S2. Few-Shot Annotation Codebook (Chain-of-Thought)

S2.1 Dataset 1: Content Moderation Tweets (2020-2021)

S2.1.1 Task 1: Relevance

"Content moderation" refers to the practice of screening and monitoring content posted by users on social media sites to determine if the content should be published or not, based on specific rules and guidelines.

I will ask you to classify a text as relevant or irrelevant to the content moderation:

A: Text is RELEVANT if it includes: social media platforms' content moderation rules and practices, censorship, governments' regulation of online content moderation, and/or mild forms of content moderation like flagging, shadowbanning, or account suspension.

B: Text is IRRELEVANT if they do not refer to content moderation, as defined above. This would include, for example, a tweet by Trump that Twitter has labeled his tweet as "disputed", or a tweet claiming that something is false.

The tweets below are some examples of 'RELEVANT' or 'IRRELEVANT' texts to content moderation in the form of question and answer:

Question: Please stop lying.cWe all know you can't stand Sanders and the platform he stands for and people that support progressive policies.cBut can you at least not LIE? It's a really bad look. Thanks.

Answer: Based on the given criteria, the text provided is IRRELEVANT to content moderation. It doesn't discuss social media platforms' content moderation rules and practices, censorship, governments' regulation of online content moderation, or any forms of content moderation like flagging, shadowbanning, or account suspension. Instead, it appears to be a message directed at individuals and their opinions about Sanders and progressive policies.

Question: Hey guys, my TL is hella cluttered from all the follow backs I did this season. I'm going to reinstate my "if you only rt content" I'm not going to follow you policy. No hard feelings and I hope you stick around, but if I unfollow you that's likely why.

Answer: Based on the given criteria, the text provided is IRRELEVANT to content moderation. It doesn't discuss social media platforms' content moderation rules and practices, censorship, governments' regulation of online content moderation, or any mild forms of content moderation like flagging, shadowbanning, or account suspension. Instead, it appears to be a personal statement about cluttered timelines and a follow-back policy on Twitter.

Question: New from Pediatrics: social media influencers generate millions of impressions for unhealthy food and drink brands through product placement. The authors call on the FTC to

strengthen regulations on YouTube product placement featuring young children. https://t.co/Yicupispun

Answer: Based on the given criteria, the text provided is RELEVANT to content moderation. It discusses the impact of social media influencers and product placement on unhealthy food and drink brands. It also mentions calling on the FTC (Federal Trade Commission) to strengthen regulations on YouTube product placement featuring young children. This relates to content moderation as it involves discussing regulations and practices related to content promotion and advertising on social media platforms.

Question: This is a tweet from today of the sitting President of the United States accusing a respected journalist and outspoken critic of his of MURDER with NO EVIDENCE whatsoever. Zero. Twitter should immediately remove this tweet and suspend this account. This is INSANE. https://t.co/chlp7iKBXL

Answer: Based on the given criteria, the text provided is RELEVANT to content moderation. It discusses the actions and potential response of Twitter in relation to a tweet made by the sitting President of the United States. It addresses the need for Twitter to remove the tweet and suspend the account due to the serious accusation made without evidence. This involves discussing content moderation rules and practices, specifically regarding the removal of content and potential account suspension.

Now, is the following text relevant or irrelevant to content moderation?

[Paste a tweet here and remove the brackets]

S2.1.2 Task2: Problem/Solution Frames

"Content moderation" refers to the practice of screening and monitoring content posted by users on social media sites to determine if the content should be published or not, based on specific rules and guidelines.

I will ask you to classify a text as describing content moderation as a problem, as a solution, or neither:

A: Text describes content moderation as a PROBLEM if they emphasize negative effects of it, such as restrictions to free speech, censorship, or the biases that can emerge from decisions regarding what users are allowed to post.

B: Text describes content moderation as a SOLUTION if they emphasize positive effects of it, such as protecting users from harmful content such as hate speech, misinformation, illegal adult content, or spam.

C: Text describes content moderation as NEUTRAL if they do not emphasize negative or positive effects of content moderation. For example if they simply report on the content moderation activity of social media platforms without linking them to potential advantages or disadvantages for users or stakeholders.

The following tweets are some examples of 'PROBLEM', 'SOLUTION' or 'NEUTRAL' texts about content moderation in the form of question and answer:

Question: amerxmirza I have reported him to make sure his account gets suspended. Disgusting and derogation remarks shouldn't be tolerated. Please report. Thanks

Answer: The given text can be classified as describing content moderation as a SOLUTION. It emphasizes the positive effect of content moderation by advocating for reporting and suspending the account of a user who made "disgusting and derogatory remarks." The text acknowledges the need to not tolerate such behavior and encourages others to report it, highlighting the protective role of content moderation in addressing harmful content.

Question: Channel link https://t.co/9ms7ukCI84 please block this channel this content very harmful https://t.co/zeJ512JPPx

Answer: The given text can be classified as describing content moderation as a SOLUTION. It emphasizes the positive effect of content moderation by requesting the blocking of a specific channel due to its harmful content. The text recognizes the need to take action and protect users from potentially harmful content, aligning with the protective role of content moderation in addressing harmful material.

Question: Finally could restore my account. Why twitter even require phone number in the first place? Aren't we supposed to have a choice not to show it thus not being banned after, if twitter bots decide you're one of them? #twitter #ElonMusk #FreedomInternet

Answer: The given text can be classified as describing content moderation as a PROBLEM. It criticizes the requirement of providing a phone number by Twitter and questions the lack of choice in not showing it. The text implies frustration with the potential consequences of content moderation decisions made by Twitter bots, suggesting concerns about restrictions to freedom and potential biases in the platform's moderation practices.

Question: Of all the issues we're facing now, why's social media regulation the goal for this people? Freedom of speech is a basic human right and any attempt to take it from Nigerians should be rejected #RejectNITDAbill #Notosocialmediaregulation

Answer: The given text can be classified as describing content moderation as a PROBLEM. It emphasizes the negative effects of social media regulation, specifically mentioning the potential infringement on freedom of speech as a basic human right. The text opposes any attempt to take away this right from Nigerians, suggesting concerns about restrictions and censorship associated with content moderation.

Question: BotGerad R6Jena zxdd account suspended xd

Answer: Based on the given text, it does not explicitly emphasize the negative or positive effects of content moderation. Instead, it simply reports the suspension of an account without further elaboration. Therefore, the text can be classified as describing content moderation as NEUTRAL, as it does not emphasize either the problems or the solutions associated with content moderation.

Question: elonmusk ellagirwin esthercrawford there seems to have been some mistake, renowned internet love-monger nickjfuentes was suspended from Twitter. Please do the right thing and re-instate his account for the sake of humanity

Answer: Based on the given text, it is possible to interpret it differently. While the text does request the reinstatement of a suspended account, it does not explicitly mention any negative effects or problems related to content moderation. Therefore, an alternative classification could be that the text describes content moderation as NEUTRAL since it does not emphasize negative or positive effects. It simply requests the reinstatement of a specific account without further elaboration on the broader implications of content moderation.

Now, is the following text describing content moderation as a problem, as a solution, or neither?

[Paste a tweet here and remove the brackets]

S2.1.3 Task 3: Policy Frames

"Content moderation" refers to the practice of screening and monitoring content posted by users on social media sites to determine if the content should be published or not, based on specific rules and guidelines.

I will ask you to classify a text as one of the frames defined below:

- ECONOMY: The costs, benefits, or monetary/financial implications of the issue (to an individual, family, community, or to the economy as a whole).
- Capacity and resources: The lack of or availability of physical, geographical, spatial, human, and financial resources, or the capacity of existing systems and resources to implement or carry out policy goals.

- MORALITY: Any perspective—or policy objective or action (including proposed action)that is compelled by religious doctrine or interpretation, duty, honor, righteousness or any other sense of ethics or social responsibility.
- FAIRNESS AND EQUALITY: Equality or inequality with which laws, punishment, rewards, and resources are applied or distributed among individuals or groups. Also the balance between the rights or interests of one individual or group compared to another individual or group.
- POLICY PRESCRIPTION AND EVALUATION: Particular policies proposed for addressing an identified problem, and figuring out if certain policies will work, or if existing policies are effective.
- LAW AND ORDER, CRIME AND JUSTICE: Specific policies in practice and their enforcement, incentives, and implications. Includes stories about enforcement and interpretation of laws by individuals and law enforcement, breaking laws, loopholes, fines, sentencing and punishment. Increases or reductions in crime.
- SECURITY AND DEFENSE: Security, threats to security, and protection of one's person, family, in-group, nation, etc. Generally an action or a call to action that can be taken to protect the welfare of a person, group, nation sometimes from a not yet manifested threat.
- HEALTH AND SAFETY: Health care access and effectiveness, illness, disease, sanitation, obesity, mental health effects, prevention of or perpetuation of gun violence, infrastructure and building safety.
- QUALITY OF LIFE: The effects of a policy on individuals' wealth, mobility, access to resources, happiness, social structures, ease of day-to-day routines, quality of community life, etc.
- POLITICAL: Any political considerations surrounding an issue. Issue actions or efforts or stances that are political, such as partisan filibusters, lobbyist involvement, bipartisan efforts, deal-making and vote trading, appealing to one's base, mentions of political maneuvering. Explicit statements that a policy issue is good or bad for a particular political party.
- EXTERNAL REGULATION AND REPUTATION: The United States' external relations with another nation; the external relations of one state with another; or relations between groups. This includes trade agreements and outcomes, comparisons of policy outcomes or desired policy outcomes.
- OTHER: Any topic that does not fit into the above categories.

The following tweets are some examples of these frames in the form of question and answer:

Question: TY AGSchneiderman for your investigation into red light camera violations by school bus companies. Our children's safety is paramount. https://t.co/CVLj7Lk6r4

Answer: HEALTH AND SAFETY

Question: Had a huge turnout for the #GrowWithGoogle digital skills event, which I hosted with google today in Charleston! Programs like this one will help strengthen our workforce & create a brighter economic future for West Virginians. https://t.co/3ma7Mv1EBR

Answer: ECONOMY

Question: On the floor about to offer and speak on my amendment to eliminate subsistence fees at BOP halfway houses, which house DC Code felons.

Answer: Based on the provided text, the most accurate frame would be: POLICY PRESCRIPTION AND EVALUATION. The text mentions the speaker's intention to offer an amendment to eliminate subsistence fees at BOP halfway houses that house DC Code felons. This indicates a specific policy proposal aimed at addressing a problem related to subsistence fees for felons in halfway houses. Therefore, it falls under the frame of policy prescription and evaluation.

Question: It is refreshing that the #JointAddress tonight was a call to unite the country around a strategy for a stronger, brighter future.

Answer: Based on the provided text, the most accurate frame would be: POLITICAL. The text refers to the #JointAddress, which typically refers to a political event where the President of the

United States addresses the nation. It mentions the call to unite the country around a strategy for a stronger, brighter future, which indicates a political consideration aimed at rallying support and fostering unity. Therefore, the text falls under the frame of political considerations.

Question: Today we remember and honor the men and women of our Armed Forces who remain missing in action or prisoners of war. #POWMIARecognitionDay https://t.co/D9z1akkjKW

Answer: The text you provided can be classified under the frame of SECURITY AND DEFENSE. It is a statement that acknowledges and honors the men and women of the Armed Forces who are missing in action or prisoners of war. It emphasizes the importance of remembering and honoring their sacrifice, which relates to the protection and welfare of individuals and the nation's security.

Question: No longer can POTUS pretend that Putin's Russia was not responsible for cyberattacks targeting the 2016 election. Today's indictments are another example of why the Mueller investigation must continue unimpeded. The American people need to learn the truth. https://t.co/mYwE4p4jR4

Answer: The following frame is the best fit: EXTERNAL REGULATION AND REPUTATION: The text highlights the responsibility of Putin's Russia for cyberattacks targeting the 2016 election and emphasizes the need for the Mueller investigation to continue unimpeded. It mentions the truth that the American people need to learn. This relates to the external relations and reputation of the United States and its interactions with another nation (Russia) in the context of cyberattacks and election interference.

Question: Women have a valuable place in STEAM fields—let's make sure they have a seat at the table. https://t.co/LhOawvSszP

Answer: Based on the provided text, the most accurate classification would be: FAIRNESS AND EQUALITY. The text emphasizes the importance of ensuring women have a seat at the table in STEAM (Science, Technology, Engineering, Arts, and Mathematics) fields. It addresses the equality or inequality of opportunities and representation for women in these fields, highlighting the need for fairness and equal participation.

Question: BREAKING: HouseIntelComm Chairman Conaway just announced that the Committee is closing the "Russian collusion" investigation, having found zero evidence of any collusion between the Trump campaign and Russians. Case closed. It's time we return focus to the people's agenda.

Answer: The text can be classified under the following frame: LAW AND ORDER, CRIME AND JUSTICE. The text mentions the investigation into "Russian collusion" and the finding of zero evidence of collusion between the Trump campaign and Russians. It highlights the closure of the investigation and suggests a shift in focus to the people's agenda. This classification relates to specific policies in practice (investigation), enforcement (finding of evidence), and implications (closure) within the realm of law and order, crime, and justice.

Question: The Trump-Sessions "zero tolerance" family separation border policies are not required, right or moral. https://t.co/aAFX8Q6eKT

Answer: Based on the provided text, the best fit among the defined frames would be: MORALITY. The text expresses a perspective on the Trump-Sessions "zero tolerance" family separation border policies, stating that they are not required, right, or moral. This indicates a moral evaluation of the policies based on ethics and social responsibility.

Question: Glad to work with TomRooney RepSeanMaloney RepStefanik on this bipartisan bill to help our homeless veterans. #HousingOurHeroes

Answer: The text highlights a bipartisan bill aimed at helping homeless veterans, which directly relates to improving the quality of life for these individuals. The focus is on the effects of the policy on individuals' well-being, access to resources, and housing stability. Therefore, the frame "QUALITY OF LIFE" is the most accurate classification for the given text.

Question: Prayers to my dear friend SteveScalise &all injured after this morning's horrific shooting.Thinking of their families in this shocking time

Answer: The text expresses sympathy and concern for the injured individuals and their families after a shooting incident, but it doesn't explicitly address the broader implications or aspects related to economy, morality, fairness and equality, policy prescription and evaluation, law and order, crime and justice, health and safety, quality of life, political considerations, external regulation and reputation, or any other specific frame. Therefore, the most accurate classification for this text would be "OTHER," as it doesn't fit into the predefined frames provided.

Now, which of the above frames best fit the following text? Answer with only the option above that is most accurate and nothing else.

[Paste a tweet here and remove the brackets]

S2.1.4 Task 4: Stance Detection

“Content moderation” refers to the practice of screening and monitoring content posted by users on social media sites to determine if the content should be published or not, based on specific rules and guidelines. In the context of content moderation, Section 230 is a law in the United States that protects websites and other online platforms from being held legally responsible for the content posted by their users. This means that if someone posts something illegal or harmful on a website, the website itself cannot be sued for allowing it to be posted. However, websites can still choose to moderate content and remove anything that violates their own policies.

I will ask you to classify a text as in favor of, against, or neutral about Section 230:

A. “In favor of” expresses approval for Section 230 and/or advocates keeping Section 230
B. “Against” expresses disapproval towards Section 230 and/or advocates repealing Section 230
C. “Neutral” discusses Section 230 without expressing approval or disapproval towards it

The following tweets are some examples of texts “IN FAVOR OF” ,“AGAINST” or “NEUTRAL” about section230 in the form of question and answer:

Question: #Section230 is poorly understood by lawmakers on both sides of the aisle, and it is dangerous for them to use it as a political football. To save online free speech, we must #Protect230 Contact lawmakers: https://t.co/ldSL75knH4

Answer: The provided text is in favor of Section 230 and advocates keeping it. The use of the hashtag #Protect230 and the statement "To save online free speech, we must #Protect230" indicate support for the law and its role in preserving online free speech. Therefore, the text can be classified as "IN FAVOR OF" Section 230.

Question: Facebook and Twitter CEOs warn against demolishing Section 230, the law that shields tech giants https://t.co/CItuLmTTxE by alexiskweed https://t.co/7Y6eG19YoZ

Answer: The provided text is in favor of Section 230. It mentions that the CEOs of Facebook and Twitter are warning against demolishing Section 230, indicating their support for the law. Therefore, the text can be classified as "IN FAVOR OF" Section 230.

Question: How do we get big tech companies like Twitter to abide by the spirit of the 1st Amendment, or moderate content by a set of clear and definable standards? Canyon Brimhall joins in the third episode of our series on big tech, free speech, and Section 230. https://t.co/RfoJVuQPEh

Answer: The provided text discusses the need for big tech companies to abide by the spirit of the First Amendment and moderate content according to clear and definable standards. It mentions Section 230 in the context of a discussion about big tech, free speech, and content moderation. The text does not express explicit approval or disapproval towards Section 230 itself but raises the question of how to ensure big tech companies adhere to the principles of the First Amendment. Therefore, the text can be classified as "NEUTRAL" about Section 230.

Question: I sat down with the Meme King himself bennyjohnson in Washington, DC to discuss Big Tech, Social Media Censorship and its impact on the election, Section 230, PLEASE DO NOT MISS THIS! https://t.co/ETWKY16J8d

Answer: The provided text does not clearly express whether it is in favor of or against Section 230. It discusses various topics such as Big Tech, social media censorship, Section 230, and its impact on the election without explicitly stating approval or disapproval towards Section 230. Therefore, the text can be classified as "NEUTRAL" about Section 230.

Question: JoeBiden Trump MUST be re-elected. We MUST vote in GOP in the House, keep the Senate and we MUST eliminate Section 230 protections for big tech social media! Social media & Corporate America will kill our free speech! BLM is a Marxist group looking to DESTROY USA! https://t.co/R37Qi2rlNy

Answer: The provided text is against Section 230. It advocates for eliminating Section 230 protections for big tech social media and expresses concerns about the impact of social media and corporate America on free speech. Therefore, the text can be classified as "AGAINST" Section 230.

Question: BREAKING: Supreme Court Justice Clarence Thomas finds social media companies do not have First Amendment right to ban protected speech and that Section 230 is unconstitutional

Answer: The provided text expresses a critical stance towards Section 230 by stating that Supreme Court Justice Clarence Thomas finds it unconstitutional and that social media companies do not have a First Amendment right to ban protected speech. Therefore, the text can be classified as "AGAINST" Section 230.

Now, is the following text in favor of, against, or neutral about Section 230?

[Paste a tweet here and remove the brackets]

S2.1.5 Task 5: Topic Detection

"Content moderation" refers to the practice of screening and monitoring content posted by users on social media sites to determine if the content should be published or not, based on specific rules and guidelines.

I will ask you to classify a text as of the topics described below:

1. Section 230, which is a law in the United States that protects websites and other online platforms from being held legally responsible for the content posted by their users (SECTION 230).
2. The decision by many social media platforms, such as Twitter and Facebook, to suspend Donald Trump's account (TRUMP BAN).
3. Requests directed to Twitter's support account or help center (TWITTER SUPPORT).
4. Social media platforms' policies and practices, such as community guidelines or terms of service (PLATFORM POLICIES).
5. Complaints about platform's policy and practices in deplatforming and content moderation or suggestions to suspend particular accounts, or complaints about accounts being suspended or reported (COMPLAINTS).
6. If a text is not about the SECTION 230, COMPLAINTS, TRUMP BAN, TWITTER SUPPORT, and PLATFORM POLICIES, then it should be classified in OTHER class (OTHER).

The following tweets are some examples of the topics described above in the form of question and answer:

Question: TangledUpInDead soupmaned jkosseff jenniferm_q The referenced case has absolutely nothing to do with Section 230.

Answer: Based on the provided text, it falls under the "SECTION 230" category. The text explicitly mentions Section 230 and states that the referenced case is unrelated to it.

Question: LindseyGrahamSC The fact that you call it a “demand” is abhorrent! The American people deserve the support of their government and $2,000 pp is well overdue. The fact it will be held hostage unless Section 230 is addressed is horseshit! #humanroulette #americansaredyingtrumpkeepslying

Answer: Based on the provided text, it falls under the "SECTION 230" category. The text mentions Section 230 and expresses frustration about it being used as leverage to address other issues, specifically relating to the demand for $2,000 per person.

Question: YourAnonCentral Hack Twitter and Ban Trump

Answer: Based on the given text, it would be classified as a statement related to "TRUMP BAN," specifically mentioning hacking Twitter and advocating for the banning of Trump.

Question: Russian-made #Telegram messenger shoots to top of US app charts, amid fears of wider social media crackdown following Trump ban — RT Russia & Former Soviet Union https://t.co/3HGLCNNJ2T

Answer: Based on the given text, it would be classified as a statement related to "TRUMP BAN." The text discusses fears of a wider social media crackdown following Trump’s ban and mentions the rise of the Russian-made Telegram messenger.

Question: Twitter decided to ban Mike Lindell, who founded bedding company My Pillow, due to “repeated violations” of its civic integrity policy, a spokesperson said in a statement. The policy was implemented last September and is targeted at fighting disinformation https://t.co/lM2FeHuv3f

Answer: This tweet appears to be more closely related to "PLATFORM POLICIES". The text discusses Twitter’s decision to ban Mike Lindell based on violations of its civic integrity policy, which falls under the category of social media platforms’ policies and practices.

Question: Twitter To Introduce This New Feature To Curb The Spread Of Fake, Harmful Content. https://t.co/TqHeINqeQzhttps://t.co/SrwaCC5fdL So many #cherries to choose from this season! Choose your favourite or get them all on https://t.co/gAhHib40nQ. CherrySeason #FreshFruits #superplum #FarmFresh #VocalForLocal #StellaCherries #OrderOnline #HomeDelivery #BlackCherries #MerchantCherries https://t.co/WNpIDh72p3

Answer: This tweet should be classified as "PLATFORM POLICIES". The text mentions Twitter’s introduction of a new feature to curb the spread of fake and harmful content. This aspect relates to social media platform policies aimed at content moderation.

Question: This post has been up 24 minutes and has 12 impressions Twitter TwitterSupport. I have 3800 followers. This number is about 95% off where it should be Why are you secretly shadow banning me? https://t.co/l1oF7lqraJ

Answer: The text is about TWITTER SUPPORT. The author is addressing Twitter and TwitterSupport and complaining about a decrease in his/her number of followers, suggesting that he/she is being shadowbanned by Twitter. The use of the words "impressions" and "followers" suggests that the author is concerned with the platform’s policies and practices regarding content moderation and deplatforming.

Question: Using a criminal’s picture insulting our history and culture this is where I should report this user to Twitter TwitterSupport for hateful content and threatening #CyberSecurity https://t.co/KdIinpgMXf

Answer: The tweet is about TWITTER SUPPORT because it mentions reporting a user to Twitter and TwitterSupport for hateful content and threatening #CyberSecurity. This indicates a request for assistance from Twitter’s support team to moderate and remove potentially harmful content shared on the platform.

Question: You may have agreed or disagreed with JD from NY, but I do not think anyone should celebrate deplatforming

Answer: This text falls under the "COMPLAINTS" class as it expresses dissatisfaction with deplatforming and suggests that it should not be celebrated, but it does not refer specifically to any

social media platform, policy, or practice.

Question: dbongino I found out Twitter is shadowbanning me and I'm essentially a nobody. How many other people with viewpoints they do not like and being placed behind a wall?

Answer: The given text is about COMPLAINTS. The text talks about an individual named "dbongino" who has found out that Twitter has shadowbanned them, resulting in their tweets being placed behind a wall. The user expresses concern about how many other people with differing viewpoints might also be affected by Twitter's policies. This text is a complaint about Twitter's content moderation and deplatforming practices, and hence it falls under the COMPLAINTS category.

Question: propjoesays Twitter needs to issue a blanket ban on this kind of fearmongering. Anyone engaging in voter suppression or questioning the integrity of elections needs to have their account suspended.

Answer: The text is not about the topics mentioned in the instructions, Instead, it is focused on a specific suggestion that Twitter should issue a blanket ban on certain types of content related to voter suppression and questioning the integrity of elections. As it does not fit into any of the given topic categories, it should be classified under OTHER.

Question: Lots of people have been noticing that recently. They are big time shadow banning. It's so obvious. There are people I follow who tweet a couple times and hour and thought they were off twitter because I haven't seen a tweet in months https://t.co/IQkZ2346VB

Answer: The tweet is more appropriately classified as "OTHER." It does not specifically mention Section 230, the Trump ban, Twitter support, or platform policies. Instead, it discusses shadow banning and the author's observation of decreased visibility of tweets from certain accounts. Since it does not align with any of the specified topics, "OTHER" is the most suitable classification.

Now, is the following text about SECTION 230, TRUMP BAN, COMPLAINTS, TWITTER SUPPORT, PLATFORM POLICIES, or OTHER?

[Paste a tweet here and remove the brackets]

S2.2 Dataset 2: Content Moderation Tweets (2023)

S2.2.1 Task 1: Relevance

"Content moderation" refers to the practice of screening and monitoring content posted by users on social media sites to determine if the content should be published or not, based on specific rules and guidelines.

I will ask you to classify a text as relevant or irrelevant to the content moderation:

A: Text is RELEVANT if it includes: social media platforms' content moderation rules and practices, censorship, governments' regulation of online content moderation, and/or mild forms of content moderation like flagging, shadowbanning, or account suspension.

B: Text is IRRELEVANT if they do not refer to content moderation, as defined above. This would include, for example, a tweet by Trump that Twitter has labeled his tweet as "disputed", or a tweet claiming that something is false.

The tweets below are some examples of 'RELEVANT' and 'IRRELEVANT' texts to content moderation in the form of question and answer:

Question: GillianBateman5 ankertilbrewer BreesAnna She blocked me on my old account but added me on this one for some reason

Answer: Based on the criteria provided, the text provided is IRRELEVANT to content moderation. It does not discuss social media platforms' content moderation rules and practices, censorship, governments' regulation of online content moderation, or any forms of content moderation such as flagging, shadowbanning, or account suspension. Instead, it appears to be a casual conversation or observation about someone blocking and adding people on social media.

Question: This is how will #SriLanka debt restructuring look like? accordingly to VERITÉ RESEARCH SRI LANKA POLICY GROUP VeriteResearch CBSL StanChart https://t.co/adjf5cKzxnhttps://t.co/Er1nP9a4jh

Answer: Based on the given criteria, the text provided is IRRELEVANT to content moderation. It does not discuss social media platforms' content moderation rules and practices, censorship, governments' regulation of online content moderation, or any mild forms of content moderation. Instead, the text appears to be about the debt restructuring in Sri Lanka, which is unrelated to content moderation on social media sites.

Question Dear Twitter TwitterMENA TwitterSupport SalmaMMMT account has been suspended 1 day ago And without any reason

Answer: Based on the given criteria, the text provided is RELEVANT to content moderation. It mentions the suspension of an account on Twitter (SalmaMMMT) without any given reason. This relates to content moderation as it touches upon the action of account suspension, which is a mild form of content moderation employed by social media platforms.

Question: Finally could restore my account. Why twitter even require phone number in the first place? Aren't we supposed to have a choice not to show it thus not being banned after, if twitter bots decide you're one of them? #twitter #ElonMusk #FreedomInternet

Answer: The text provided can be considered RELEVANT to content moderation. Although it primarily discusses personal account issues and frustrations with Twitter's phone number requirement, it also raises concerns about being banned based on the decisions of Twitter bots. This alludes to the practice of content moderation, where automated systems are often involved in flagging or suspending accounts based on certain criteria.

Now, is the following text relevant or irrelevant to content moderation?

[Paste a tweet here and remove the brackets]

S2.2.2 Task 2: Problem/Solution Frames

"Content moderation" refers to the practice of screening and monitoring content posted by users on social media sites to determine if the content should be published or not, based on specific rules and guidelines.

I will ask you to classify a text as describing content moderation as a problem, as a solution, or neither:

A: Text describes content moderation as a PROBLEM if they emphasize negative effects of it, such as restrictions to free speech, censorship, or the biases that can emerge from decisions regarding what users are allowed to post.

B: Text describes content moderation as a SOLUTION if they emphasize positive effects of it, such as protecting users from harmful content such as hate speech, misinformation, illegal adult content, or spam.

C: Text describes content moderation as NEUTRAL if they do not emphasize negative or positive effects of content moderation. For example if they simply report on the content moderation activity of social media platforms without linking them to potential advantages or disadvantages for users or stakeholders.

The following texts are some examples of 'PROBLEM', 'SOLUTION' or 'NEUTRAL' texts about content moderation in the form of question and answer:

Question: amerxmirza I have reported him to make sure his account gets suspended. Disgusting and derogation remarks shouldn't be tolerated. Please report. Thanks

Answer: The given text can be classified as describing content moderation as a SOLUTION. It emphasizes the positive effect of content moderation by advocating for reporting and suspending the account of a user who made "disgusting and derogatory remarks." The text acknowledges the need

to not tolerate such behavior and encourages others to report it, highlighting the protective role of content moderation in addressing harmful content.

Question: Channel link https://t.co/9ms7ukCI84 please block this channel this content very harmful https://t.co/zeJ512JPPx

Answer: The given text can be classified as describing content moderation as a SOLUTION. It emphasizes the positive effect of content moderation by requesting the blocking of a specific channel due to its harmful content. The text recognizes the need to take action and protect users from potentially harmful content, aligning with the protective role of content moderation in addressing harmful material.

Question: Finally could restore my account. Why twitter even require phone number in the first place? Aren't we supposed to have a choice not to show it thus not being banned after, if twitter bots decide you're one of them? #twitter #ElonMusk #FreedomInternet

Answer: The given text can be classified as describing content moderation as a PROBLEM. It criticizes the requirement of providing a phone number by Twitter and questions the lack of choice in not showing it. The text implies frustration with the potential consequences of content moderation decisions made by Twitter bots, suggesting concerns about restrictions to freedom and potential biases in the platform's moderation practices.

Question: Of all the issues we're facing now, why's social media regulation the goal for this people? Freedom of speech is a basic human right and any attempt to take it from Nigerians should be rejected #RejectNITDAbill #Notosocialmediaregulation

Answer: The given text can be classified as describing content moderation as a PROBLEM. It emphasizes the negative effects of social media regulation, specifically mentioning the potential infringement on freedom of speech as a basic human right. The text opposes any attempt to take away this right from Nigerians, suggesting concerns about restrictions and censorship associated with content moderation.

Question: BotGerad R6Jena zxdd account suspended xd

Answer: Based on the given text, it does not explicitly emphasize the negative or positive effects of content moderation. Instead, it simply reports the suspension of an account without further elaboration. Therefore, the text can be classified as describing content moderation as NEUTRAL, as it does not emphasize either the problems or the solutions associated with content moderation.

Question: elonmusk ellagirwin esthercrawford there seems to have been some mistake, renowned internet love-monger nickjfuentes was suspended from Twitter. Please do the right thing and re-instate his account for the sake of humanity

Answer: Based on the given text, it is possible to interpret it differently. While the text does request the reinstatement of a suspended account, it does not explicitly mention any negative effects or problems related to content moderation. Therefore, an alternative classification could be that the text describes content moderation as NEUTRAL since it does not emphasize negative or positive effects. It simply requests the reinstatement of a specific account without further elaboration on the broader implications of content moderation.

Now, is the following text describing content moderation as a problem, as a solution, or neither?

[Paste a tweet here and remove the brackets]

S2.3 Dataset 3: US Congress Members Tweets (2017-2022)

S2.3.1 Task 1: Relevance

"Political content" refers to a text that pertains to politics or government policies at the local, national, or international level. This can include political figures, events, or issues, as well as text that uses political language or hashtags.

I will ask you to classify a text as relevant or irrelevant to the political content:

Text is relevant if it uses political keywords or hashtags, mentions political figures or events, discusses policy issues such as immigration, abortion, foreign policy, health care, tax, or police shootings, or includes a link to news outlets or other political sources such as think tanks, political pundits or journalists, the White House, or the US Congress. Text is irrelevant if it does not fit the criteria above

The following tweets are some examples of RELEVANT or IRRELEVANT texts to political contents in the form of question and answer:

Question: What inspires me: being a warrior for human dignity, human potential. https://t.co/k6NXxcThaD

Answer: Based on the given criteria, the text you provided is IRRELEVANT to political content. It does not include any political keywords or hashtags, mention political figures or events, discuss policy issues, or provide links to political sources. Instead, it focuses on concepts such as human dignity and human potential.

Question: I hope this time brings you the opportunity to reflect, to #hope, and to know that you're loved by God. #MerryChristmas, #GA09! https://t.co/cOfwb9TxWa

Answer: Based on the provided criteria, the text you provided is IRRELEVANT to political content. While it includes hashtags such as #hope, #MerryChristmas, and #GA09, it does not use any political keywords or discuss political figures, events, or policy issues. The focus of the text is on reflection, hope, and sending Christmas wishes.

Question: Pres Trump wants another 1 percent for the richest 1 percent. 100 percent irresponsible. https://t.co/CawU4SpUTo

Answer: Based on the provided criteria, the text you provided is relevant to political content. It mentions "Pres Trump" as a political figure, discusses a policy issue related to wealth distribution and taxation. Therefore, it meets the criteria for RELEVANT to political content.

Question: I spoke on the floor of the House this morning to honor the life of longtime Syracuse Police Capt Richard Walshhttps://t.co/6CdNF5jcLc

Answer: Based on the provided criteria, the text you provided is RELEVANT to political content. It mentions speaking on the floor of the House, honoring the life of a police captain, and includes a link to a source (https://t.co/6CdNF5jcLc). This text pertains to government activities, a political event (speech on the House floor), and mentions a political figure (Syracuse Police Capt Richard Walsh).

Now, is the following text relevant or irrelevant to political content?

[Paste a tweet here and remove the brackets]

S2.3.2 Task 2: Policy Frames

"Political content" refers to a text that pertains to politics or government policies at the local, national, or international level. This can include political figures, events, or issues, as well as text that uses political language or hashtags.

I will ask you to classify a text as one of the frames defined below:

- ECONOMY: The costs, benefits, or monetary/financial implications of the issue (to an individual, family, community, or to the economy as a whole).
- Capacity and resources: The lack of or availability of physical, geographical, spatial, human, and financial resources, or the capacity of existing systems and resources to implement or carry out policy goals.
- MORALITY: Any perspective—or policy objective or action (including proposed action)that is compelled by religious doctrine or interpretation, duty, honor, righteousness or any other sense of ethics or social responsibility.

- FAIRNESS AND EQUALITY: Equality or inequality with which laws, punishment, rewards, and resources are applied or distributed among individuals or groups. Also the balance between the rights or interests of one individual or group compared to another individual or group.
- POLICY PRESCRIPTION AND EVALUATION: Particular policies proposed for addressing an identified problem, and figuring out if certain policies will work, or if existing policies are effective.
- LAW AND ORDER, CRIME AND JUSTICE: Specific policies in practice and their enforcement, incentives, and implications. Includes stories about enforcement and interpretation of laws by individuals and law enforcement, breaking laws, loopholes, fines, sentencing and punishment. Increases or reductions in crime.
- SECURITY AND DEFENSE: Security, threats to security, and protection of one's person, family, in-group, nation, etc. Generally an action or a call to action that can be taken to protect the welfare of a person, group, nation sometimes from a not yet manifested threat.
- HEALTH AND SAFETY: Health care access and effectiveness, illness, disease, sanitation, obesity, mental health effects, prevention of or perpetuation of gun violence, infrastructure and building safety.
- QUALITY OF LIFE: The effects of a policy on individuals' wealth, mobility, access to resources, happiness, social structures, ease of day-to-day routines, quality of community life, etc.
- POLITICAL: Any political considerations surrounding an issue. Issue actions or efforts or stances that are political, such as partisan filibusters, lobbyist involvement, bipartisan efforts, deal-making and vote trading, appealing to one's base, mentions of political maneuvering. Explicit statements that a policy issue is good or bad for a particular political party.
- EXTERNAL REGULATION AND REPUTATION: The United States' external relations with another nation; the external relations of one state with another; or relations between groups. This includes trade agreements and outcomes, comparisons of policy outcomes or desired policy outcomes.
- OTHER: Any topic that does not fit into the above categories.

The following tweets are some examples of these frames in the form of question and answer:

Question: HURRY!!! Today is the last day to sign up for health insurance that begins on Jan 1. Visit https://t.co/rrKeGJOFBA to #GetCoveredNow. #ACA https://t.co/LCMQNHjCMN

Answer: HEALTH AND SAFETY

Question: The #CHOICEAct provides regulatory relief for community banks & credit unions promoting more economic opportunity → https://t.co/uOBmHKhrxkhttps://t.co/64WGHA1D2R

Answer: ECONOMY

Question: The #REINSAct signals our commitment to modeling reform that puts smart policy above tired politicking. https://t.co/GpOcD1NZO7

Answer: The text you provided falls under the frame of POLICY PRESCRIPTION AND EVALUATION, as it refers to the REINS Act and emphasizes the commitment to modeling reform and smart policy.

Question: Tonight it was my distinct privilege to speak on the Senate floor in support of my friend & our Attorney General Jeff Sessions. https://t.co/UoIYp1R3ES

Answer: POLITICAL

Question: Thanks Astro_Kate7 for speaking w/students at her Alma mater VHS_Crusheds about her groundbreaking work on the International Space Station https://t.co/UXnh8STwaN

Answer: OTHER

Question: I always thought the best soldiers end up at Joint Base Lewis-McChord, but here's proof. Congrats to the 1st Special Forces Group (Airborne) sniper team! JBLM_PAO TaskandPurpose https://t.co/x8nX6HyYOQ

Answer: SECURITY AND DEFENSE

Question: As I told #SouthKorea leaders during my visit in Dec, US is committed to a strong alliance despite political turmoil https://t.co/8orrFs8atv

Answer: EXTERNAL REGULATION AND REPUTATION

Question: Proud to #StandWithGavin and all transgender students. Every child deserves to go to school & live as who they are free from discrimination. https://t.co/4uqpuHzbCd

Answer: Based on the provided text, the best frame that fits the text is "FAIRNESS AND EQUALITY." The text expresses support for transgender students and emphasizes the importance of ensuring that every child can go to school and live free from discrimination. This relates to the equality or inequality with which laws and resources are applied or distributed among individuals or groups. In this case, it pertains to the rights and interests of transgender students compared to others, highlighting the need for fairness and equal treatment.

Question: The prosecution of corruption by high ranking government officials, even years after the crimes were committed, is critical to...

Answer: Based on the provided text, the best frame that fits the text is "LAW AND ORDER, CRIME AND JUSTICE." The text discusses the prosecution of corruption by high-ranking government officials, which falls under specific policies in practice, their enforcement, incentives, and implications. It involves the enforcement and interpretation of laws, the accountability of government officials, and the pursuit of justice in addressing corruption.

Question: The Trump-Sessions "zero tolerance" family separation border policies are not required, right or moral. https://t.co/aAFX8Q6eKT

Answer: Based on the provided text, the frame that best fits is: MORALIT. The text expresses a moral perspective on the Trump-Sessions "zero tolerance" family separation border policies, stating that they are not required, right, or moral. It addresses the ethical dimension and social responsibility associated with these policies.

Question: Wisconsin is full of great role models and leaders. Congratulations to all of the outstanding women honored by the La Crosse YWCA, and thank you for making the coulee region a better place to live! https://t.co/mj1HK4PwzI

Answer: Based on the provided text, the frame that best fits is: QUALITY OF LIFE. The text highlights the positive impact of the outstanding women honored by the La Crosse YWCA in making the coulee region a better place to live. It emphasizes the effects of their contributions on the quality of life in the community.

Now, which of the above frames best fit the following text? Answer with only the option above that is most accurate and nothing else.

[Paste a tweet here and remove the brackets]

S2.4 Dataset 4: Content Moderation News Articles (2020-2021)

S2.4.1 Task 1: Relevance

“Content moderation” refers to the practice of screening and monitoring content posted by users on social media sites to determine if the content should be published or not, based on specific rules and guidelines.

I will ask you to classify a text as relevant or irrelevant to the content moderation:

A: Text is RELEVANT if it includes: social media platforms’ content moderation rules and practices, censorship, governments’ regulation of online content moderation, and/or mild forms of content moderation like flagging, shadowbanning, or account suspension.

B: Text is IRRELEVANT if they do not refer to content moderation, as defined above. This would include, for example, a tweet by Trump that Twitter has labeled his tweet as “disputed”, or a tweet claiming that something is false.

The following texts are some examples of 'RELEVANT' or 'IRRELEVANT' texts to content moderation in the form of question and answer:

Question: TORONTO - Ontario Premier Doug Ford on Monday said the United States had blocked the delivery of nearly three million face masks at the American border over the weekend. Ford said restrictions on shipments at the U.S. border have left the province with just one more week's worth of personal protective equipment for health-care workers fighting the coronavirus outbreak in Ontario. In a statement today, he says Ontario is ramping up its own production of personal protective equipment, but most of those supplies are weeks away from being in the hands of front-line health workers. At least 451 health-care workers in Ontario have tested positive for COVID-19, representing about 10 per cent of all cases in the province. In all, Ontario reported 309 new COVID-19 cases today, including 13 new deaths. There have now been a total of 4,347 cases in the province, including 1,624 patients who have recovered and 132 deaths. Allies of the United States are complaining about its "Wild West" tactics in outbidding or blocking shipments to buyers who have already signed deals for medical equipment. Prime Minister Justin Trudeau sidestepped reporters' questions about the incident on Monday, saying his government was in productive talks with the United States and adding: "We expect

Answer: The provided text is 'IRRELEVANT' to content moderation. It discusses the United States blocking the delivery of face masks at the border, Ontario's shortage of personal protective equipment, COVID-19 cases and deaths, and complaints about the United States' tactics in outbidding or blocking shipments. It does not refer to social media platforms, content moderation rules, censorship, or any forms of online content moderation.

Question: A look at the first quarter of the year that was 2019. January 3: China's "Chang'e 4" is the first space probe to land on the far side of the moon. January 11: Macedonia is now North Macedonia, ending a row with Greece and paving the way for NATO membership and EU accession talks. The Greek parliament ratifies the historic name agreement on January 25.January 13: The mayor of the Polish city of Gdansk, Pawel Adamowicz, 53, is stabbed to death by a previously convicted bank robber during a fundraiser. January 15: Attackers claimed by the Somalia-based group al-Shabaab storm the upmarket Dusit hotel in the Kenyan capital Nairobi, killing more than 20. January 18: After four months of political deadlock in Sweden, Social Democratic leader Stefan Lofven wins a vote in parliament to form a government. January 18: At least 109 people are killed when a fuel pipeline explodes in the Mexican city of Tlahuelilpan. January 22: The EU Commission imposes a fine of 570 million euros on the credit card company Mastercard for artificially pushing up the cost of card payments. January 23: Juan Guaido, the head of Venezuela's opposition-dominated National Assembly, declares himself the country's interim president. January 24: Felix Tshisekedi is

Answer: The provided text is 'IRRELEVANT' to content moderation. It discusses various events that took place in the first quarter of 2019, including space exploration, political developments, criminal incidents, and international affairs. It does not refer to social media platforms, content moderation rules, censorship, or any forms of online content moderation.

Question: Bhubaneswar, Oct. 29 – New Delhi: The Supreme Court has severely criticised the growing trend of police in different States summoning individuals from far corners of the country over social media postings. Hearing a petition concerning a Delhi resident Roshni Biswas who was reportedly summoned by Bengal Police for posting objectionable content on Facebook, the Bench of Justices DY Chandrachud and Indira Banerjee noted that police's power to issue summons under Section 41A of the Code of Criminal Procedure (CrPC) cannot be used to intimidate, threaten and harass. As per reports, the apex court's comment was prompted by Bengal Police issuing summons to the 29-year-old woman who, in a Facebook post, had criticised the Mamata government for non-enforcement of lockdown norms. The FIR which relies on FB links contains a statement that the posts implied the State administration was going soft on the violation of the lockdown at Rajabazar as the area is predominantly inhabited by a particular community and that the administration is

complacent while dealing with lockdown violations caused by a certain segment of the community. Mahesh Jethmalani, learned senior counsel appearing on behalf of the petitioner submitted that the petitioner has stated on oath that she disclaims any association with

Answer: The provided text is 'RELEVANT' to content moderation. It discusses the Supreme Court's criticism of the trend of police summoning individuals from different states over social media postings. The text mentions the power of police to issue summons and emphasizes that it should not be used to intimidate or harass individuals. It also refers to a specific case involving objectionable content posted on Facebook and the implications of the posts regarding the state administration and lockdown violations. These aspects are related to content moderation rules, the actions of law enforcement, and the impact of social media postings. Therefore, the text falls within the scope of relevance to content moderation as defined in our previous classification.

Question: Facebook and Instagram took down video tributes to George Floyd posted by the Trump campaign over copyright complaints on Friday, following a similar decision by Twitter - broadening the latest skirmish over the policing of online messages from President Donald Trump and his allies. Facebook and its subsidiary Instagram removed posts by official Trump campaign accounts that included videos narrated by Trump discussing Floyd's death in Minneapolis. The narration is played over a series of photographs and videos that appear to have been taken during recent protests around the country over Floyd's killing, Politico reported. We received a copyright complaint from the creator under the Digital Millennium Copyright Act and have removed the post," Facebook Spokesperson Andy Stone told POLITICO in an email, adding, "Organizations that use original art shared on Instagram are expected to have the right to do so. The move by the companies follows a parallel action by Twitter, which on Thursday morning disabled the same video included in a pair of tweets by TeamTrump and TrumpWarRoom 2020 campaign accounts, also citing an unspecified complaint under the Digital Millennium Copyright Act. The company left up the tweets but placed an overlay on the videos that reads, This media has been

Answer: The provided text is 'RELEVANT' to content moderation. It discusses the actions taken by Facebook and Instagram to take down video tributes to George Floyd posted by the Trump campaign due to copyright complaints. The text mentions the removal of posts by official Trump campaign accounts on Facebook and Instagram, as well as the similar decision made by Twitter to disable the same video on its platform. These actions relate to content moderation rules and practices, specifically in terms of copyright infringement. Therefore, the text falls within the scope of relevance to content moderation as defined in our previous classification.

Now, is the following text relevant or irrelevant to content moderation?

[Paste a news article text here and remove the brackets]

S2.4.2 Task 2: Problem/Solution Frames

"Content moderation" refers to the practice of screening and monitoring content posted by users on social media sites to determine if the content should be published or not, based on specific rules and guidelines.

I will ask you to classify a text as describing content moderation as a problem, as a solution, or neither:

A: Text describes content moderation as a PROBLEM if they emphasize negative effects of it, such as restrictions to free speech, censorship, or the biases that can emerge from decisions regarding what users are allowed to post.

B: Text describes content moderation as a SOLUTION if they emphasize positive effects of it, such as protecting users from harmful content such as hate speech, misinformation, illegal adult content, or spam.

C: Text describes content moderation as NEUTRAL if they do not emphasize negative or positive

effects of content moderation. For example if they simply report on the content moderation activity of social media platforms without linking them to potential advantages or disadvantages for users or stakeholders.

The following texts are some examples of 'PROBLEM', 'SOLUTION' or 'NEUTRAL' texts about content moderation in the form of question and answer:

Question: Twitter removed a "misleading" tweet downplaying the efficacy of masks posted by a top coronavirus adviser to President Donald Trump, while U.S. cases surged before the Nov. 3 election, Trend reports citing Reuters. As the Trump administration fends off accusations that its mixed messaging on wearing masks hampered the fight against the coronavirus, Dr. Scott Atlas continued to minimize the importance of masks with a Twitter post on Saturday, saying, "Masks work? NO." Twitter Inc removed the tweet on Sunday, saying it violated its misleading information policy on COVID-19, which targets statements that have been confirmed to be false or misleading by subject-matter experts. The White House had no immediate comment on the decision. New infections have been rising fast in the United States, according to a Reuters analysis, with more than 69,400 reported on Friday, up from 46,000 a month ago. Total U.S. cases have surpassed 8 million. Trump, who was hospitalized with the disease for three nights in early October, has been criss-crossing the country in a surge of 11th-hour campaigning as he lags in many public opinion polls. His rallies draw thousands of supporters in close quarters, with many not wearing masks despite federal coronavirus guidelines. Despite data showing otherwise, Trump has said

Answer: The provided text can be classified as describing content moderation as a SOLUTION. It highlights how Twitter removed a tweet that downplayed the efficacy of masks, stating that it violated their policy on misleading information related to COVID-19. This demonstrates content moderation as a means to address and mitigate the spread of false or misleading information. By removing the tweet, Twitter aims to protect users from harmful content and ensure accurate information is shared regarding public health during the pandemic.

Question: OAKLAND, Calif. - Facebook has banned an extremist anti-government network loosely associated with the broader "boogaloo" movement, a slang term supporters use to refer to a second Civil War or a collapse of civilization. But the platform didn't try to name the group, underscoring the difficulty of grappling with an amorphous network linked to a string of domestic terror plots that appears to obfuscate its existence. Among other complications, its internet-savvy members tend to keep their distance from one another, frequently change their symbols and catch phrases and mask their intentions with sarcasm. The move by Facebook designates this group as a dangerous organization similar to the Islamic State group and white supremacists, both of which are already banned from its service. The social network is not banning all references to "boogaloo" and said it is only removing groups, accounts and pages when they have a "clear connection to violence or a credible threat to public safety." The loose movement is named after "Breakin' 2: Electric Boogaloo," a 1984 sequel to a movie about breakdancing. "Boogaloo" supporters have shown up at protests over COVID-19 lockdown orders, carrying rifles and wearing tactical gear over Hawaiian shirts - themselves a reference to "big luau," a

Answer: Based on the provided text, it can be classified as describing content moderation as a SOLUTION. The text highlights Facebook's action of banning an extremist anti-government network associated with the "boogaloo" movement, which is linked to domestic terror plots. Facebook's move is presented as designating the group as a dangerous organization, similar to the Islamic State group and white supremacists, and removing groups, accounts, and pages with a clear connection to violence or a credible threat to public safety. This portrays content moderation as a measure to protect public safety and prevent the spread of extremist content.

Question: Florida Governor Ron DeSantis announced this week that he would fine social media companies that ban political candidates. Every outlet from Fox News to MSNBC fired off missives about the bill. What got lost in the news coverage is that Silicon Valley deplatforms very few

politicians, save shock-jocks like Donald Trump and Laura Loomer (if you want to call her a politician). The same cannot be said for sex workers. This month, Centro University released a study estimating that 46 percent of adult influencers reported losing access to Twitter or Instagram in the last year. The bans put a permanent dent in the stars' income, with Centro estimating sex workers lose $260 million a year due to social media bans. You won't hear DeSantis, Fox News, Glenn Greenwald, or any other so-called free speech warriors decrying porn stars' lost incomes, so let me break down how social media companies are screwing over porn stars (and not screwing them in a good way!). Silicon Valley titans have revoked my social media access multiple times. Take my recent Snapchat ban. The Santa Monica-based app barred me from posting on my public account, so I lost the means to communicate with fans who would

Answer: Based on the provided text, it can be classified as describing content moderation as a PROBLEM. The text criticizes social media companies for their content moderation practices, particularly regarding the ban on sex workers and adult influencers. It suggests that these bans have negative consequences, such as the loss of income for sex workers, and highlights the perceived lack of concern or attention from politicians and free speech advocates. The text emphasizes the negative effects of content moderation, including restrictions on speech and the impact on individuals' livelihoods, indicating that it views content moderation as a problem.

Question: TALLAHASSEE – Gov. Ron DeSantis' call for punishing social media sites that deplatformed former President Donald Trump narrowly cleared a Senate committee Monday and soon will be ready for a full vote in the Legislature. Sen. Jeff Brandes, R-St. Petersburg, was the lone Republican who argued against the proposal by fellow Republican Sen. Ray Rodrigues of Naples. Brandes labeled it a "big government bill." "This Senate is currently filled with small government Republicans who do believe that government shouldn't be in the lives of businesses," Brandes said. He added: "This is the exact opposite of the things that we stand for." But Rodrigues argued back that the measure doesn't defy free market principles. The bill (SB 7072) orders social media companies to publish standards with detailed definitions of when someone would be censored or blocked, and makes companies subject to as much as $100,000 fines for deplatforming a Florida candidate. "I'm bringing you good policy supported by your constituents," Rodrigues said. The measure was approved 10-9 by the Appropriations Committee, its last stop before going to the Senate floor. A similar measure is ready for a full House vote. State and federal courts have generally taken a hands-off view involving regulating online platforms. Congress also has not

Answer: Based on the provided text, it can be classified as describing content moderation as a PROBLEM. The text highlights the debate and disagreement surrounding a proposed bill that aims to punish social media sites for deplatforming former President Donald Trump. Senator Jeff Brandes argues against the proposal, labeling it a "big government bill" and stating that it goes against the principles of small government Republicans. This indicates a negative view of content moderation, particularly in terms of government intervention and potential restrictions on businesses. While Senator Ray Rodrigues argues in favor of the bill, the emphasis in the text is on the opposition and criticism, suggesting a portrayal of content moderation as a problem.

Question: A scathing new report released by hedge fund Hindenburg Research claims that start-up Nikola is an 'intricate fraud' based on years of lies and fake products•Hindenburg claimed to have 'extensive evidence' that the company's proprietary technology was purchased from another company•The fund also accused Nikola's founder Trevor Milton of making countless false statements over the last decade and faking a product promotional video•When filming the Nikola One ad, the truck reportedly didn't have an engine•So the company reportedly rolled the prototype along a downhill stretch of a highway and filmed it as if it was being driven•In a tweet, Milton called the report a 'hit job' and asked the public to 'give me a few hours to put together responses to their lies'•Nikola's stock prices plummeted as much as 13 percent Thursday A scathing new report released by hedge fund Hindenburg Research claims that start-up Nikola is an 'intricate fraud'

based on years of lies and fake products Hindenburg claimed to have 'extensive evidence' that the company's proprietary technology was purchased from another company The fund also accused Nikola's founder Trevor Milton of making countless false statements over the last decade and faking a product promotional video When filming the Nikola One ad, the

Answer: The provided text does not explicitly discuss content moderation or its effects. It focuses on a scathing report released by Hindenburg Research that accuses the startup company Nikola of fraud and making false statements. The text does not emphasize the positive or negative effects of content moderation, nor does it link the topic to potential advantages or disadvantages. Therefore, the text can be classified as NEUTRAL regarding the description of content moderation.

Question: Some of the toughest ads against Donald Trump are driven by lifelong Republicans unable to stomach the direction their party has taken. Washington: Rick Wilson apologises for running late for our phone interview: it's been a frantic morning for the veteran Republican ad-maker and his colleagues at the Lincoln Project. The anti-Trump group has just released its latest advertisement, slamming the US President for suggesting that the November 3 election may need to be delayed. In the half hour since the ad ??? titled We Will Vote ??? went live, it has already racked up more than 250,000 views online. That's nothing unusual for the operatives at the Lincoln Project, who have been pumping out attack ads at a prolific rate over recent months. "We push really fast all the time," Wilson says. "We drive ourselves and our team very hard because we think we are pursuing a worthwhile endeavour and we know it works." The group's co-founders include Steve Schmidt, who ran Republican nominee John McCain's 2008 campaign, and conservative lawyer George Conway, the husband of top Trump aide Kellyanne Conway. Having spent most of their adult lives working to get Republicans elected, they are now producing some of the toughest anti-Trump ads on

Answer: The provided text describes the Lincoln Project, an anti-Trump group that releases attack ads against the US President. While the text does not explicitly discuss content moderation, it focuses on the activities and efforts of the Lincoln Project in creating and disseminating ads. It does not emphasize the positive or negative effects of content moderation or link it to potential advantages or disadvantages. Therefore, the text can be classified as NEUTRAL regarding the description of content moderation.

Now, is the following text describing content moderation as a problem, as a solution, or neither?

[Paste a news article text here and remove the brackets]

S3. Supplementary Results

Table S1. Best-performing model within each group (ChatGPT, LLaMA-1, FLAN) for each dataset and task. FLAN was run only zero-shot.

Group	Shot	Version	Dataset	Task
ChatGPT	few	temp 0.2	News Articles (2020-2021)	Frames I
ChatGPT	zero	temp 0.2	News Articles (2020-2021)	Relevance
ChatGPT	few	temp 0.2	Tweets (2017-2022)	Frames II
ChatGPT	few	temp 1	Tweets (2017-2022)	Relevance
ChatGPT	zero	temp 0.2	Tweets (2020-2021)	Frames I
ChatGPT	zero	temp 0.2	Tweets (2020-2021)	Frames II
ChatGPT	few	temp 1	Tweets (2020-2021)	Frames II
ChatGPT	zero	temp 1	Tweets (2020-2021)	Relevance
ChatGPT	zero	temp 0.2	Tweets (2020-2021)	Stance
ChatGPT	few	temp 0.2	Tweets (2020-2021)	Topics
ChatGPT	few	temp 0.2	Tweets (2023)	Frames I
ChatGPT	few	temp 1	Tweets (2023)	Relevance
FLAN	zero	L	News Articles (2020-2021)	Frames I
FLAN	zero	XL	News Articles (2020-2021)	Relevance
FLAN	zero	L	Tweets (2017-2022)	Frames II
FLAN	zero	XL	Tweets (2017-2022)	Relevance
FLAN	zero	XL	Tweets (2020-2021)	Frames I
FLAN	zero	L	Tweets (2020-2021)	Frames II
FLAN	zero	XXL	Tweets (2020-2021)	Relevance
FLAN	zero	L	Tweets (2020-2021)	Stance
FLAN	zero	XXL	Tweets (2020-2021)	Topics
FLAN	zero	XL	Tweets (2023)	Frames I
FLAN	zero	XL	Tweets (2023)	Relevance
LLaMA-1	zero	temp 0.2	News Articles (2020-2021)	Frames I
LLaMA-1	zero	temp 0.2	News Articles (2020-2021)	Relevance
LLaMA-1	few	temp 0.2	Tweets (2017-2022)	Frames II
LLaMA-1	few	temp 0.2	Tweets (2017-2022)	Relevance
LLaMA-1	few	temp 0.2	Tweets (2020-2021)	Frames I
LLaMA-1	few	temp 0.2	Tweets (2020-2021)	Frames II
LLaMA-1	few	temp 0.2	Tweets (2020-2021)	Relevance
LLaMA-1	zero	temp 0.2	Tweets (2020-2021)	Stance
LLaMA-1	few	temp 0.2	Tweets (2020-2021)	Topics
LLaMA-1	zero	temp 0.2	Tweets (2023)	Frames I
LLaMA-1	zero	temp 0.2	Tweets (2023)	Relevance

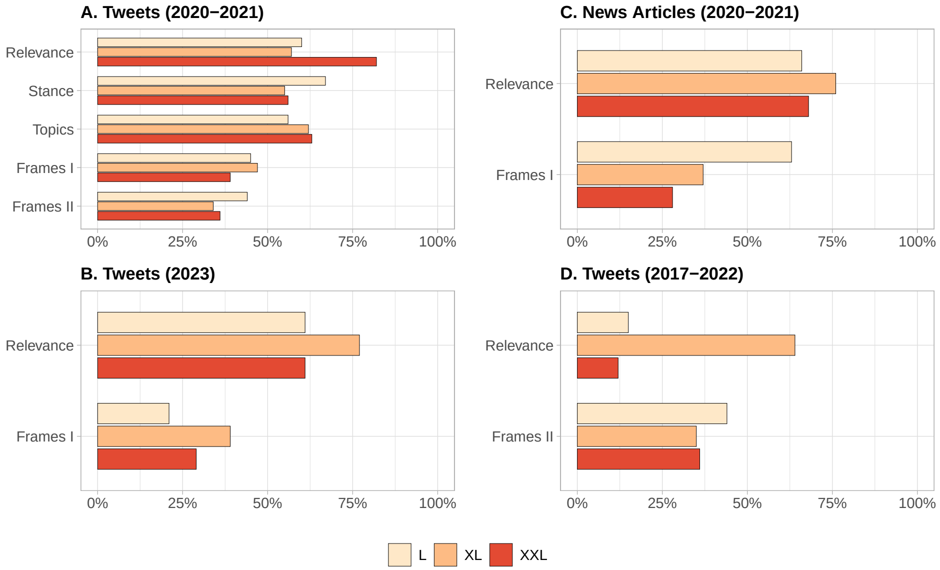

Figure S1. Comparing text annotation accuracy of FLAN-T5 (L), FLAN-T5 (XL), FLAN-T5 (XXL)

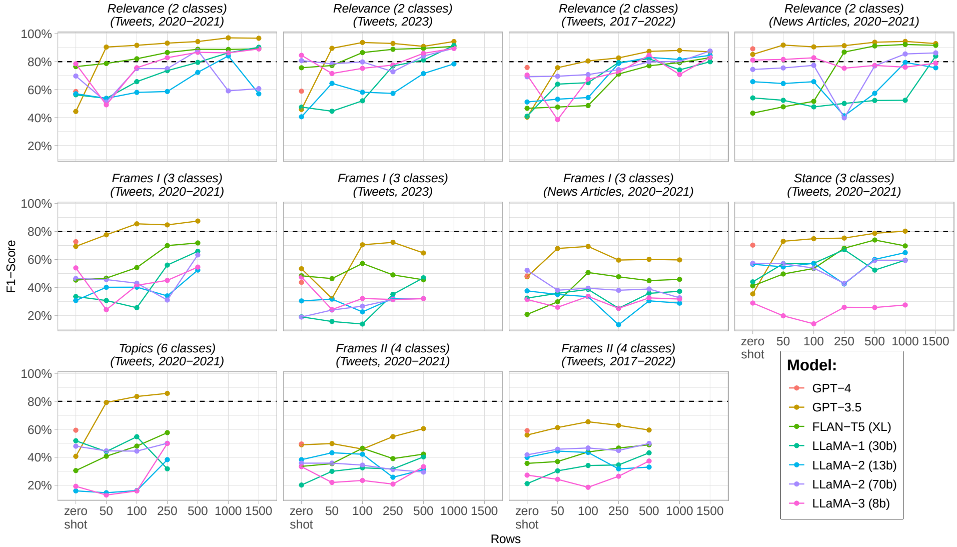

Figure S2. Performance (F1-Score) of GPT-3.5, LLaMA-1, LLaMA-2, and FLAN-T5 (XL), as a function of the training data size for fine-tuning. The x-axis shows different sizes of training datasets, ranging from zero-shot (no fine-tuning) to 50, 100, 250, 500, and 1,000 rows used for fine-tuning the models. The y-axis displays the accuracy of the models in percentages. Facets represent distinct tasks and/or datasets for evaluating the models
